# Increased Retention of Litter‐Derived Organic Carbon With Increasing Initial Carbon Content in Temperate Agricultural Soils

**DOI:** 10.1111/gcb.70646

**Published:** 2025-12-08

**Authors:** Neha Begill, Steffen A. Schweizer, Axel Don, Carmen Hoeschen, Marcus Schiedung, Georg Guggenberger, Christopher Poeplau

**Affiliations:** ^1^ Thünen Institute of Climate‐Smart Agriculture Braunschweig Germany; ^2^ Chair of Soil Science, TUM School of Life Sciences Technical University of Munich Freising Germany; ^3^ Institute of Earth System Sciences, Section Soil Science Leibniz Universität Hannover Hannover Germany

**Keywords:** ^13^C labeling, incubation, mineral‐associated organic carbon, particle size fractionation, saturation deficit, SOC loading

## Abstract

Stabilized soil organic carbon (SOC) accrual plays a crucial role in long‐term atmospheric CO_2_ sequestration. The organic carbon in the fine silt and clay size fraction (OC_fine_) is typically mineral‐associated and thus relatively stable. However, the SOC saturation concept suggests that the OC_fine_ has limited capacity for additional carbon (C) storage, thereby constraining further C sequestration. Low‐OC and fine‐textured soils are thought to have greater potential to stabilize additional OC than High‐OC and coarse‐textured soils due to their higher available storage space. Here, we assessed soils' potential to stabilize additional OC using 21 temperate agricultural soils, varying in SOC (0.7%–10.2%), silt + clay content (32%–92%), and OC loading of fine fraction (17–135 g C kg^−1^). We investigated the decomposition and recovery of uniform ^13^C labeled litter after 2 years in two size‐based fractions: OC_coarse_ (> 20 μm, the OC associated with coarse silt and sand) and OC_fine_ (< 20 μm). Litter‐derived OC retention increased significantly with initial SOC content and fine fraction OC loading, primarily driven by the OC_coarse_ fraction, which indicated that less added C was utilized by microbes when enough C was already abundant. In contrast, litter‐derived OC_fine_ formation was negatively correlated with initial SOC and fine fraction OC loading. However, when normalized to the amount of actually decomposed litter, initial SOC and texture did not significantly affect the efficiency of OC_fine_ formation. NanoSIMS showed litter‐derived OC forming at distinct microscale patches, partly overlapping with OM‐ and mineral‐dominated sites. Both findings together revealed that initial SOC content in the studied range, OC loading of the fine fraction, or even soil texture may not be major limiting factors of new OC_fine_ formation. Instead, increasing initial SOC content appeared to have a positive effect on litter‐derived OC retention by retarding its mineralization.

## Graphical abstract

This study examined how much new carbon soils can store by evaluating 21 agricultural soils that ranged from low to high organic carbon. We found that soils already rich in organic carbon retained more of the added plant‐derived carbon because microbes decomposed less of it, and that mineral surfaces did not limit the formation of new stable carbon. These findings show that even carbon‐rich soils can continue to build long‐lasting organic carbon, highlighting their importance as carbon sinks in a changing climate.

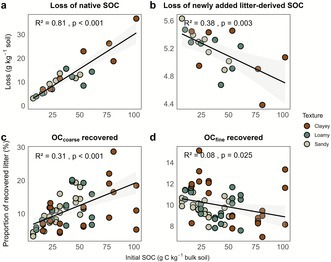

## 1 Introduction

Soil organic carbon (SOC) formation is fundamental for many soil functions and climate regulation. Therefore, increasing SOC stocks not only enhances soil fertility and sustains agricultural productivity, but can also contribute to climate change mitigation (Minasny et al. 2017; UNFCCC 2015). Achieving these benefits depends on stabilizing SOC for the long term, which is largely governed by mineral associations and microbial interactions (Lehmann et al. 2020; Schmidt et al. 2011). The capacity of soils to stabilize additional organic carbon (OC) was often evaluated through the C saturation concept, which considers the limits/capacities set by the amount of fine silt and clay sized minerals (Cotrufo et al. 2019; Hassink 1997). This means higher SOC contents would reduce free mineral surfaces, thereby limiting further stabilization (Kaiser and Guggenberger 2003). This stable SOC is primarily linked with mineral‐associated OC in the fine fraction (OC_fine_), through direct interactions with mineral surfaces or physical protection in micropores and aggregates, effectively shielding it from microbial decomposition (Kögel‐Knabner et al. 2008; Lehmann and Kleber 2015; Totsche et al. 2018). On the other hand, coarse particulate organic carbon (OC_coarse_) is supposed to decompose faster by being more susceptible to microbial mineralization (Lavallee et al. 2020). However, except when OC_coarse_ is occluded within soil aggregates, it becomes physically protected from microbes and decomposes more slowly (Angst et al. 2023).

The saturation deficit of soils is often defined as the difference between the current OC_fine_ content and its theoretical maximum OC_fine_. According to this, soils with already high SOC content (per fine particle content) have less potential to form new OC_fine_ (Stewart et al. 2008). Estimates by Georgiou et al. (2022) suggested a maximum observed OC loading of approximately 86 g C kg^−1^ silt and clay size fraction for high activity clays (2:1). At the same time, the authors concluded that globally almost all soils are strongly undersaturated. Cropland was reported to have an average mineralogical OC saturation of only 31% ± 2%, which is significantly lower than natural/less‐managed (forest and grassland) with an average OC saturation of 46% ± 3%. Nevertheless, the authors also found a negative correlation of initial SOC content and C sequestration efficiency, potentially suggesting that soils approaching their maximum storage capacity might lose the ability to stabilize new C (Stewart et al. 2008). However, the literature on the importance of initial SOC is contradicting. A recent study by Heinemann et al. (2024) found that adding very high doses of manure did not lower the OC retention compared to lower doses in four different long‐term experiments. Across sites, the OC retention efficiency was even positively correlated to initial SOC content or the OC loading of the fine fraction. Existing literature hampers the conclusion about whether the efficiency of OC stabilization decreases with increasing OC loading of the fine fraction and whether soils truly lose their capacity to form any more OC_fine_ beyond a certain threshold. From farm to national scale, such knowledge is needed to inform decision makers which soils to focus on for SOC sequestration.

The long‐term fate of freshly added carbon (C) inputs in soils may not only depend on existing SOC contents but also on the efficiency of microbial processing and stabilization (Cotrufo et al. 2013). In High‐OC soils, microbial communities are often well adapted due to a legacy of sustained C inputs, leading to increased microbial activity and enzyme production, which can trigger the breakdown of native SOC stimulated by fresh inputs (Guenet et al. 2018; Kuzyakov et al. 2000). However, nutrient limitation, such as nitrogen, may constrain microbial efficiency in these soils (Manzoni and Cotrufo 2024). Despite this, the microbial necromass resulting from turnover is considered a major source of stable fraction (OC_fine_), which is central to long‐term SOC sequestration (Cotrufo et al. 2013; Liang et al. 2017). In contrast, low‐SOC soils are suggested to offer unoccupied mineral surfaces for potential stabilization (Six et al. 2002). These systems are typically SOC limited, leading to lower microbial residue production and greater respiration losses (Manzoni and Cotrufo 2024; Wu et al. 2024). Though priming can also occur, the long‐term stabilization of fresh OC is less effective due to limited microbial transformation and weaker spatial protection (Inagaki et al. 2020; Lehmann et al. 2020). This dual role of microbes, as agents of both SOC loss and stabilization, is known as the SOC dilemma (Janzen 2006). Sanderman et al. (2017) illustrated this complexity by showing that increased C inputs can simultaneously accelerate SOC turnover and enhance SOC storage. While focusing more on long‐term dynamics, Schiedung et al. (2023) found that microbial adaptation with increasing SOC contents over time improved stabilization of fresh OC. Despite advances in understanding SOC dynamics, predicting the fate of fresh OC across soils with different SOC contents remains a major challenge, underscoring major gaps in our knowledge.

Beside the direct organo–mineral interaction, organo–organo interactions play a significant role in forming new OC_fine_ (Possinger et al. 2020). The advance of micro‐spectroscopic and micro‐spectrometric techniques has enabled insights into the heterogeneous and piled‐up arrangement of organic matter (OM) in OC_fine_ (Schweizer 2022). Nanoscale secondary ion mass spectrometry (NanoSIMS), by resolving ^13^C‐labeled litter at biologically relevant scales, enables differentiating the spatial distribution of litter‐derived and native SOC across mineral surfaces (Wilpiszeski et al. 2019). It has been shown that litter‐derived SOC is preferentially retained at native OM patches and develops in successive spatial patterns with increasing coverage and connectivity of the patches over time (Schweizer et al. 2018; Vogel et al. 2014). The partial co‐location of litter‐derived SOC with native SOC patches and mineral surfaces suggests that besides mineral surface availability and reactivity, organic surfaces and microbial activity can be important determinants, as new OC is co‐located with preexisting native SOC patches (Wilhelm et al. 2022). The observed patchy‐distributed patterns of SOC at the microscale to some extent question the applicability of a universal upper limit for OC loading of fine fraction across all soils with similar clay mineralogy. Consequently, a better understanding of the microscale distribution of SOC across a representative dataset of agricultural topsoils will be essential to refine our understanding of organo–mineral interactions driving SOC storage and formation.

In this study, we aimed at evaluating how SOC content and initial OC loading of fine fraction affect the fate of newly added ^13^C‐labeled barley litter across a large gradient of SOC (0.7%–10.2%), a wide silt + clay content range (32%–92%), and OC loading of fine fraction (17–135 g C kg^−1^ fine fraction). Here, OC loadings refer to the amount of OC present in the fine soil fraction (silt + clay), expressed as g C kg^−1^ fraction. We examined the proportion of added labeled litter recovered as OC_fine_ and OC_coarse_, as well as the overall OC loss over time after 2 years of incubation. Additionally, we used high‐resolution nanoscale secondary ion mass spectrometry (NanoSIMS) to identify organo‐mineral and organo–organo interactions as stabilization mechanisms along the OC loading and texture gradients.

## 2 Material and Methods

### 2.1 Soil Samples Selection

**FIGURE 1:**
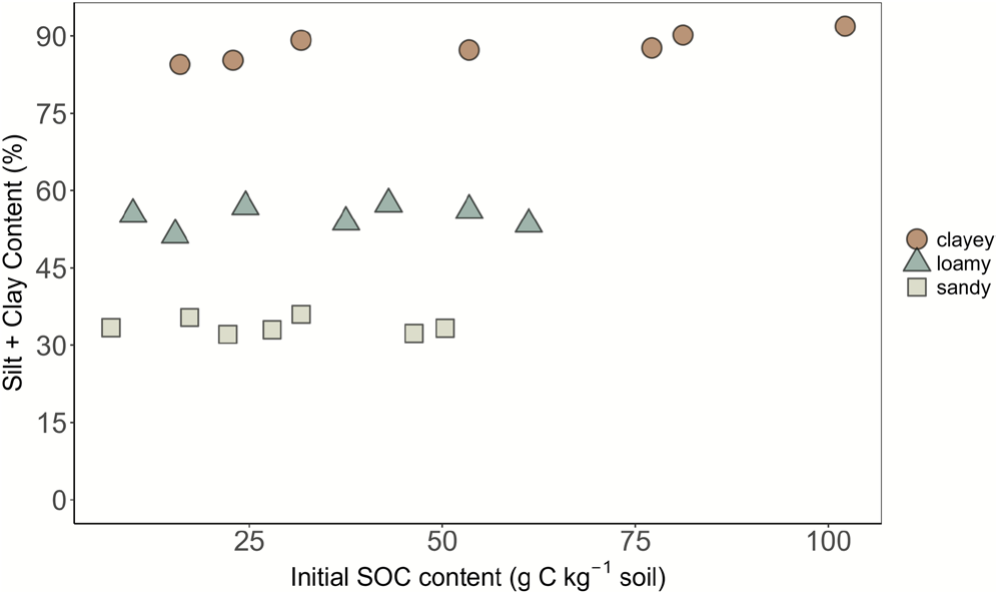
Initial soil organic carbon (SOC) content along the total clay and silt content for the selected 21 soils for the soil classes of clayey, loamy, and sandy soils based on 3104 sites from the German Agricultural Soil Inventory (more info in Table S1).

A total of 21 soil samples (Figure 1) were selected from the archive of the first German Agricultural Soil Inventory, containing 3104 sites (see Table S1 & Figure S8; Poeplau et al. 2020). The selected soils (topsoils 0–10 cm) represent a large gradient of SOC content (0.7%–10.2%) and silt + clay content (32%–92%). To ensure this variability was captured, soils were first grouped into three texture classes based on their silt + clay content (particles < 63 μm): clayey (85%–92% silt + clay), loamy (51%–57% silt + clay), and sandy (31%–36% silt + clay). From each class, seven samples were systematically and randomly selected to span the full observed range of SOC contents, ensuring similar SOC gradients across the texture classes (Figure 1).

### 2.2 Closed‐Jar Incubation Experiment

The incubation was started in December 2021 and terminated in December 2023. For each of the selected and sieved (2 mm) archive samples, triplicate subsamples of 10 g were placed in airtight glass jars (250 mL), and an equal amount of 6.5 g litter‐OC kg^−1^ soil of ^13^C‐labeled barley litter (8.06 ± 0.1 atom %) was added to each jar. The added litter had a total OC content of 45.2% and a nitrogen content of 0.8%, yielding a carbon‐to‐nitrogen ratio of 56. Before addition, the dry litter was ground to < 1 mm and thoroughly mixed into the samples to ensure homogeneous distribution in the soil. Assuming a bulk density of 1.3 and an incorporation depth of 30 cm, the added C would have resembled approximately 25 Mg C ha^−1^. Control samples (*n* = 1), without any added litter, were also prepared for each of the 21 soils. For the treated samples, the soil and litter mixture were then incubated at a controlled temperature of 20°C for 2 years. We added ammonium nitrate (0.07 g per 10 mL) to adjust for potential nitrogen limitations and stimulate early microbial activity, thereby supporting decomposition. The incubated soil samples (including control) were monitored monthly by briefly opening the jars for approximately 5 min to prevent excessive CO_2_ buildup, thoroughly mixing the soil‐litter mixture, and resealing them. Prior to litter addition, soil water‐holding capacity was adjusted to 60% and maintained throughout the experiment by monthly visual assessment and manual rewetting to ensure consistent moisture levels. Water‐holding capacity was quantified by soaking 10 g soil placed on a cotton wool‐padded funnel with water. The water content quantified when water runoff stopped was assumed to represent 100% WHC (Schroeder et al. 2021). At the end of the 2 years, the samples were dried at 60°C until completely dry for further analyses.

### 2.3 Fractionation of Soil Samples

Soil samples were fractionated before and after incubation. After drying the samples at 60°C, approximately 0.5–1 g was set aside for bulk SOC analysis, and the remaining ~9 g was immersed in 150 mL of deionized water, followed by ultrasonic dispersion at 100 JmL^−1^, as described by (Just et al. 2021). After ultrasonic dispersion, the soil solution was poured onto the 20 μm sieve, and to ensure complete separation, a continuous flow of water was directed onto the sieve until the collected water was clear. The material remaining on the sieve that was > 20 μm was collected as the coarse fraction (OC_coarse_). The remaining fine fraction (OC_fine_) suspension, < 20 μm, was treated with 0.8 g/L of CaCl_2_ as a flocculating agent. This suspension was then centrifuged at 4000 rpm for 15 min, and supernatant water was discharged. Both the coarse and fine fractions were dried at 60°C until completely dry and milled. Average mass recovery was 99% ± 0.01% with a minimum recovery of 91%. Average OC recovery in sandy, loamy, and clayey soils was 90% ± 0.07%, 91% ± 0.09%, and 87% ± 0.06%, respectively. We corrected this loss by attributing it to every fraction equally. It is unlikely that new OC was differently affected than old OC, and therefore, we consider the uncertainty due to this OC loss as negligible.

### 2.4 Microscale Analysis by NanoSIMS

To determine the distribution of barley litter‐derived ^13^C, organic matter (OM) and mineral surfaces at biologically relevant scale, nanoscale secondary ion mass spectrometry (NanoSIMS 50 L, Cameca, Gennevilliers, France) was used to analyze the fine fraction < 20 μm after 2 years of incubation. Six of the incubated samples were chosen to represent soils with low and high OC loadings across sandy, loamy, and clayey textures (more info in Table S2). Given the extensive analytical effort, it was not possible to analyze the full gradients. The described representative subset was selected to capture the effects of SOC loadings across different textures. The controls from the same soils were used to determine the elemental and isotopic baseline in the OC_fine_ to distinguish litter derived OC from native OC in the samples with ^13^C litter. Soil particles were mounted on silicon wafers, and 5 regions of interest per sample, each measuring 30 × 30 μm, were selected using scanning electron microscopy containing fine‐scale organo‐mineral associations (SEM; Jeol JSM 5900LV, Tokyo, Japan) at 2 kV. Each region was raster‐scanned with a Cs^+^ beam at an ion impact energy of 16 keV and a beam spot size of ~150 nm. Secondary ions of ^16^O−, ^12^C_2_−, ^13^C^12^C−, ^12^C^14^N−, ^28^Si−, ^27^Al^16^O−, and ^0056^Fe^16^O− were detected. The NanoSIMS instrument was calibrated for high mass resolution to distinguish between isobars such as ^13^C^−^ and ^12^C^1^H^−^. Data were recorded with a dwell time of 1 ms per pixel using a 256 × 256‐pixel grid across a 30 × 30 μm area, spanning 40 planes per scan. Raw data were corrected for electron multiplier dead time, drift, and all planes were accumulated in one single plane, using the OpenMIMS plugin in FIJI software (Gormanns et al. 2012).

The resulting NanoSIMS data were processed using multichannel machine‐learning segmentation (Inagaki et al. 2020). In a first step, this approach enabled identifying mineral‐dominated regions (high in ^16^O−) and OM‐dominated regions (high in ^12^C_2_− and ^12^C^14^N−). The area proportion of OM‐dominated surfaces by mineral‐ and OM‐dominated surfaces provided a proxy of the OM coverage. In a second step, the segmentations were applied to the ^13^C enrichment (^13^C^12^C−/(^12^C_2_− + ^13^C^12^C−); scaled to 0%–10%) in order to determine the hotspots of litter‐derived SOC. The segmentation maps were then combined to quantify the spatial overlap of ^13^C‐enriched hotspots derived from the added litter co‐located with either mineral‐dominated or OM‐dominated surfaces as elaborated in (Wilhelm et al. 2022). To account for variability in particle sizes, data were normalized to the total imaged particle area. A total of 19,842 μm^2^ of particle surface was measured (on average 2400 μm^2^ for the ^13^C‐labeled samples).

### 2.5 Carbon and Isotopic Analyses

General soil parameters (pH, EC, soil texture, initial SOC, and total nitrogen) were obtained from the First German Agricultural Soil Inventory (Poeplau et al. 2020). The soil pH was measured in a 1:2 soil‐to‐distilled water suspension, texture was determined via sieving and sedimentation, and SOC and total N were analyzed by dry combustion (Leco TruMac and RC612). For further details, see Poeplau et al. (2020). Total inorganic carbon was absent, consistent with the pH range of the soils (pH (CaCl_2_): 4.3–6.7). To quantify the incorporation of litter recovered as OC into the soil fractions and bulk soil, we measured the atomic fraction of ^13^C using an isotope ratio mass spectrometer (DeltaPlus, Thermo Fisher Scientific, Waltham, MA, USA) coupled to an elemental analyzer (CE Instruments FLASH EA 1122 NA 1500, Wigan, UK).

### 2.6 Recovery of Labeled Litter‐Derived OC

To determine how much of the added ^13^C‐labeled litter was incorporated into SOC fractions (OC_coarse_ and OC_fine_), we applied a two‐pool mixing model following Balesdent et al. (1987), using ^13^C atom %:
(1)

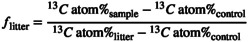

where ^*13*^ *C atom% (sample)* is the ^13^C atom percent of fractions (Bulk SOC/OC_coarse_/OC_fine_) from litter addition treatments, ^*13*^ *C atom% (control)* is the ^13^C atom percent of fractions (Bulk SOC/OC_coarse_/OC_fine_) from soils incubated without litter addition, and ^*13*^ *C atom% (litter)* is the ^13^C atom percent of added barley litter with 8.06 ± 0.1 atom%. This allowed to separate the preexisting native OC from the litter‐derived OC in the fractions and on the bulk soils, by multiplying the f(litter) with the total measured OC.

### 2.7 Litter Stabilization Efficiency

To test litter stabilization efficiency, we calculated the proportion of decomposed litter that was actually transformed as OC_fine_ using the following formula:

(2) $$\text{Litter stabilization efficiency}\;\left(\%\right)=\frac{\mathrm{New}\;{\mathrm{OC}}_{\mathrm{fine}}}{\mathrm{Litter added}-\mathrm{New}\;{\mathrm{OC}}_{\mathrm{coarse}}}\times 100\%$$

where *litter added* is the total added amount of ^13^C‐labeled litter OC, which was equal for each soil and *New OC*_*fine*_ and *New OC*_*coarse*_ are the absolute amounts of litter‐derived OC in the fine and coarse fractions expressed as [g OC g^−1^ soil]. This indicator is 100% if all added litter is recovered as OC_coarse_ or OC_fine_. The indicator declines if litter is decomposed and respired and not recovered in the fractions. It takes into account the different degrees of decomposition and transformation of the added litter (e.g., it remained in its initial state when it is in OC_coarse_) in different soils after the fixed incubation time of 2 years.

### 2.8 Statistical Analysis

Statistical analyses were conducted using R version 4.1.1 (R Core Team 2020) with the ggplot2 (Wickham 2011) package for visualization. Linear regression models were employed to examine the effects of initial SOC (or OC loading) and texture on new OC recovery from added litter. Specifically, the model assessed the interaction between initial SOC and texture and their combined impact on SOC dynamics.

Assumptions of normality and homogeneity of variance in residuals were tested using the “bptest” function from the “lmtest” package to check for heteroskedasticity (Zeileis and Hothorn 2002). If the assumptions were met, a linear model (lm) was applied. However, when heteroskedasticity was detected, Generalized Least Squares (GLS) models were used with the “gls” function from the “nlme” package (Pinheiro and Bates 1995). To account for nonconstant variance, a power variance function (“varPower”) was incorporated, ensuring robust estimation of model parameters.

## 3 Results

### 3.1 Dynamics of Old Carbon Loss in Relation to Initial SOC Across Soil Textures

**FIGURE 2:**
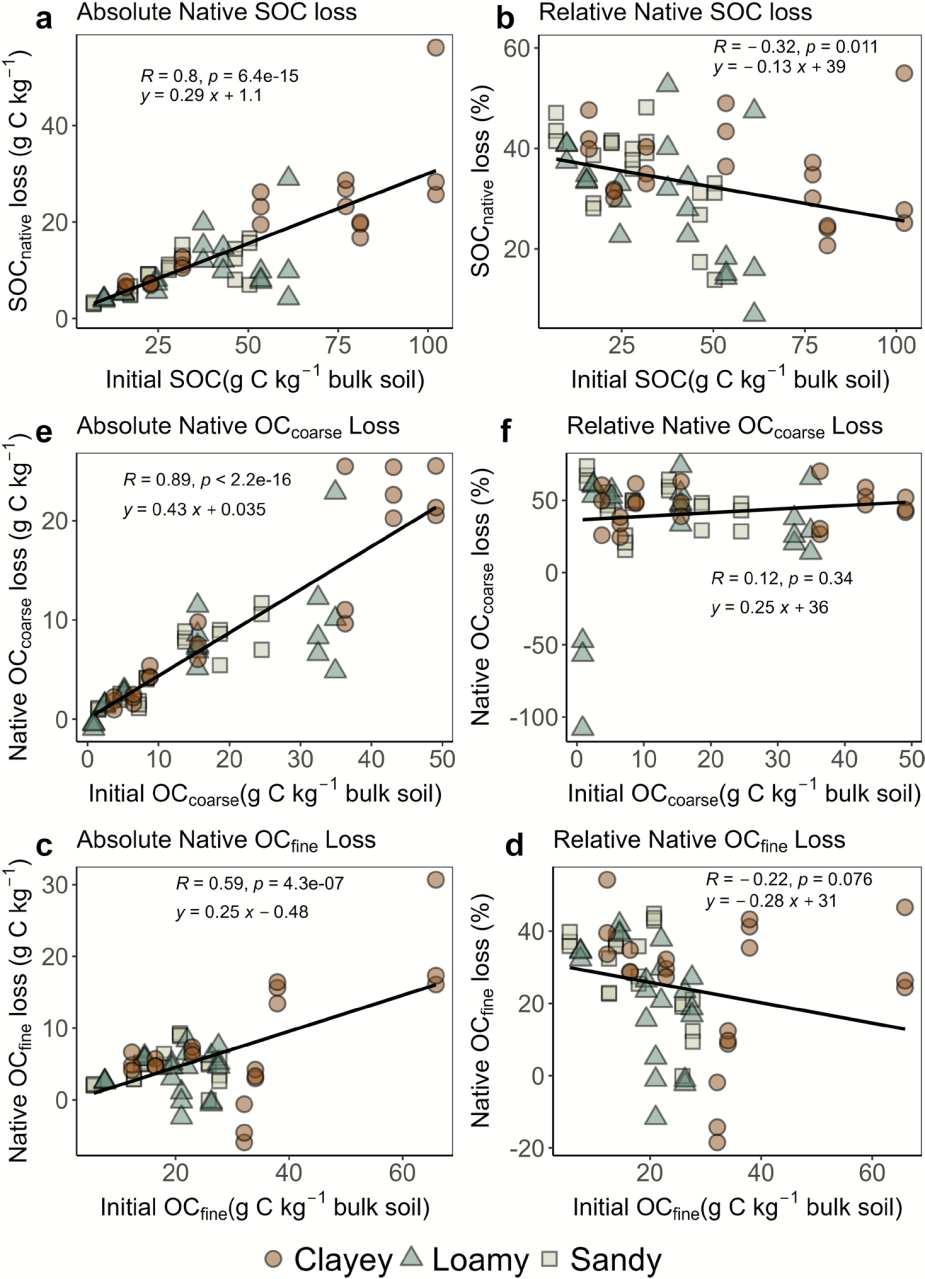
Shows absolute and relative losses of native SOC in relation to initial bulk SOC (a, b), absolute and relative losses of native OC_fine_ in relation to initial OC_fine_ (c, d), and absolute and relative losses of native OC_coarse_ in relation to initial OC_coarse_ (e, f). Positive values indicate loss; negative values indicate gain. These apparent gains may result from minor formation processes or analytical uncertainty.

After 2 years of incubation, we found that soils with higher initial SOC experienced greater absolute losses of native SOC, indicated by a significant positive correlation (*p* < 0.001) between SOC_native_ losses and initial SOC (Figure 2a). Clayey soils showed significantly higher absolute SOC_native_ losses compared to loamy and sandy soils (*p* = 0.04). In relative terms, native SOC loss was also negatively correlated with initial SOC (*p* = 0.011), suggesting proportionally smaller losses at higher initial SOC content (Figure 2b). Loamy soils had significantly lower (*p* = 0.006) relative losses compared to sandy and clayey soils.

For native OC_fine_, soils with higher initial OC_fine_ experienced greater absolute losses (*p* < 0.001; Figure 2c). Here, soil texture had no significant effect on absolute OC_fine_ losses (*p* = 0.4). Relative native OC_fine_ losses were negatively correlated with initial OC_fine_ concentrations (*p* = 0.005), although soil texture did not significantly influence these relative losses (Figure 2d). Similarly, the controls showed similar responses, and litter addition did not significantly influence SOC_native_ (*p* = 0.3) and native OC_fine_ dynamics (*p* = 0.9; Figure S1). For native OC_coarse_, absolute losses increased strongly with initial OC_coarse_ (Figure 2e; *p* < 0.001), whereas relative losses showed no significant relationship (Figure 2f; *p* > 0.05). A few samples showed apparent gains in both OC_fine_ as well as OC_coarse_ after 2 years. Apparent increases in OC_fine_ could in principle result from the decomposition of OC_coarse_ and subsequent association with fine particles. However, these small increases likely reflect minor formation processes or analytical uncertainty. Their magnitude was rather low and unrelated to initial OC loading or soil texture (data not shown), suggesting no systematic trend across soils. Native SOC losses were also not linked to the initial OC_fine_/OC_coarse_ ratio (Figure S9).

### 3.2 Litter‐Derived OC in Bulk Soils and Fractions

**FIGURE 3:**
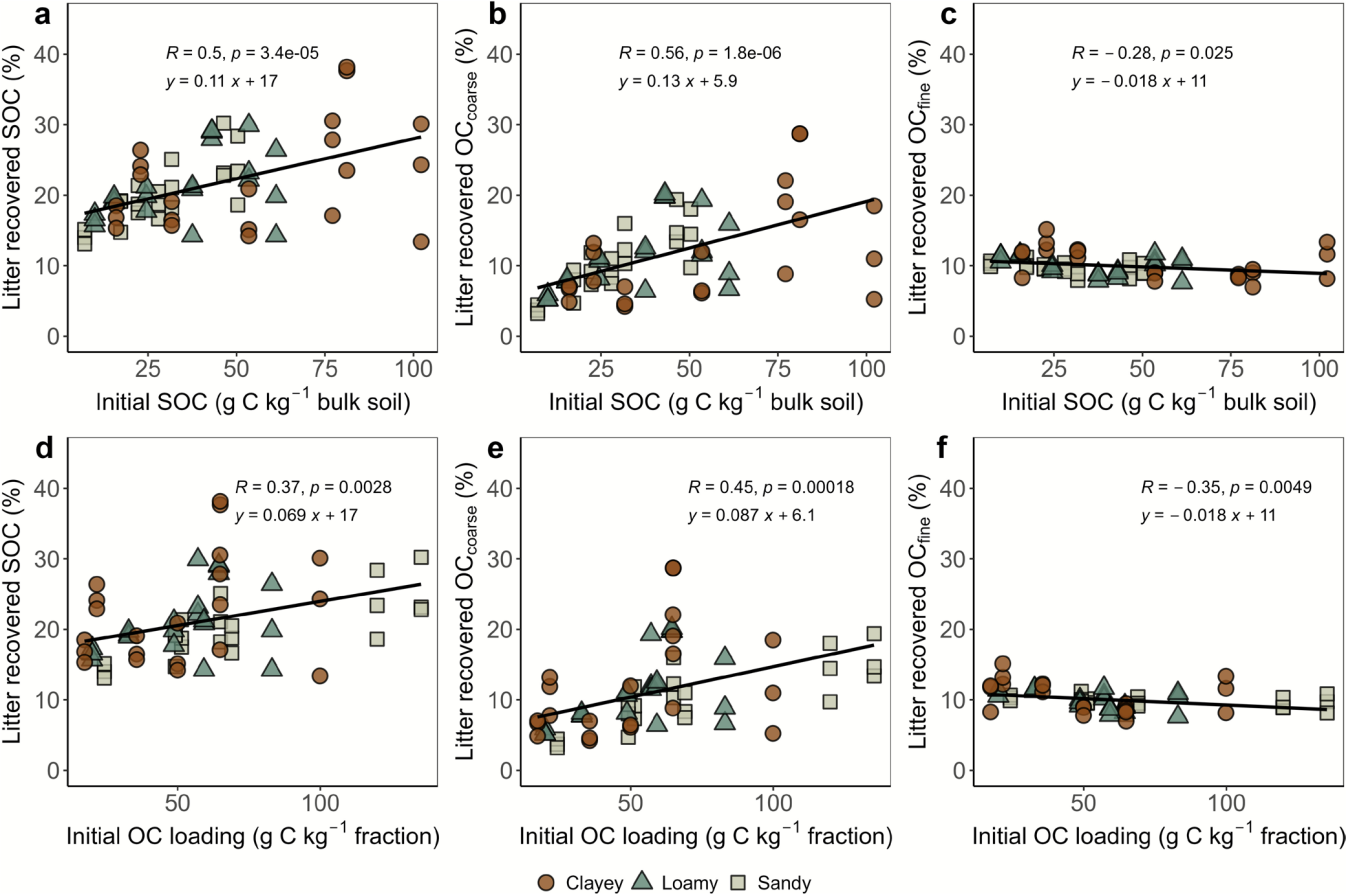
Relationships between recovered litter‐derived organic carbon (OC) in bulk soil, coarse fraction organic carbon (OC_coarse_), and fine fraction organic carbon (OC_fine_) with initial soil organic carbon (SOC) (g C kg^−1^ bulk soil) (a–c) and organic carbon (OC) loading (g C kg^−1^ silt + clay) of fine fraction (d–f) across different soil texture classes (clayey, loamy, and sandy). The litter recovered OC_fine_ is also shown in Figure S2 with individual scale.

The recovery of litter‐derived OC was on average 21% after 2 years and increased significantly with initial SOC for the bulk soil (Figure 3a and Table S3). This was primarily driven by OC_coarse_ formation that showed increasing litter‐derived OC with increasing initial bulk SOC (Figure 3b and Table S3). The OC_fine_ showed the opposite trend with decreasing litter‐derived OC with increasing initial bulk SOC (Figure 3c and Table S3). Although the highest observed recovery of litter‐derived OC in bulk soils occurred in clayey soils (13%–38% litter‐derived OC recovery), followed by loamy (14%–30%) and sandy soils (13%–30%), these differences were not statistically significant (*p* = 0.1). Similarly, texture had no significant effect in the litter‐derived OC_coarse_ formation (3%–29% litter‐derived OC recovery). In contrast, litter‐derived OC_fine_ formation showed a significant texture effect (*p* = 0.004), with higher recovery in clayey soils (7%–15% litter‐derived OC recovery) compared to loamy and sandy soils (8%–12% litter‐derived OC recovery), between which no significant differences were detected.

The relationship between litter‐derived OC recovery in bulk soils and fractions and the initial OC loading of the fine fraction followed the same pattern as that of initial SOC (Figure 3d–f). Among soil textures, sandy soils had the highest range of fine fraction OC loading (24–135 g C kg^−1^ fine fraction), followed by loamy (20–83 g C kg^−1^ fraction), with clayey soils having the lowest (17–100 g C kg^−1^ silt + clay). Soils with higher fine fraction OC loading showed a significant positive relationship with litter‐derived OC recovery in bulk soil (Figure 3d). The litter‐derived OC recovery increased in the OC_coarse_ and significantly decreased in the OC_fine_ with an increasing OC loading of the fine fraction (Figure 3f). Soil texture had no significant impact on litter‐derived OC recovery in the fine fraction (*p* = 0.1), indicating that the total amount of fine fraction (e.g., greater fine fraction in clay soils compared to sandy soils) did not drive new litter‐derived OC_fine_ recovery relative to initial OC loading (Figure 3f). The results indicate that new litter‐derived OC was allocated to the OC_fine_ even with high initial fine fraction OC loadings (Figure 3f). The OC_fine_ formation from litter‐derived OC showed a significant negative relationship with initial OC loading in the fine fraction (*p* = 0.005), indicating reduced formation at higher initial loadings.

### 3.3 Stabilization Efficiency of Added Litter in New OC_fine_ Formation

**FIGURE 4:**
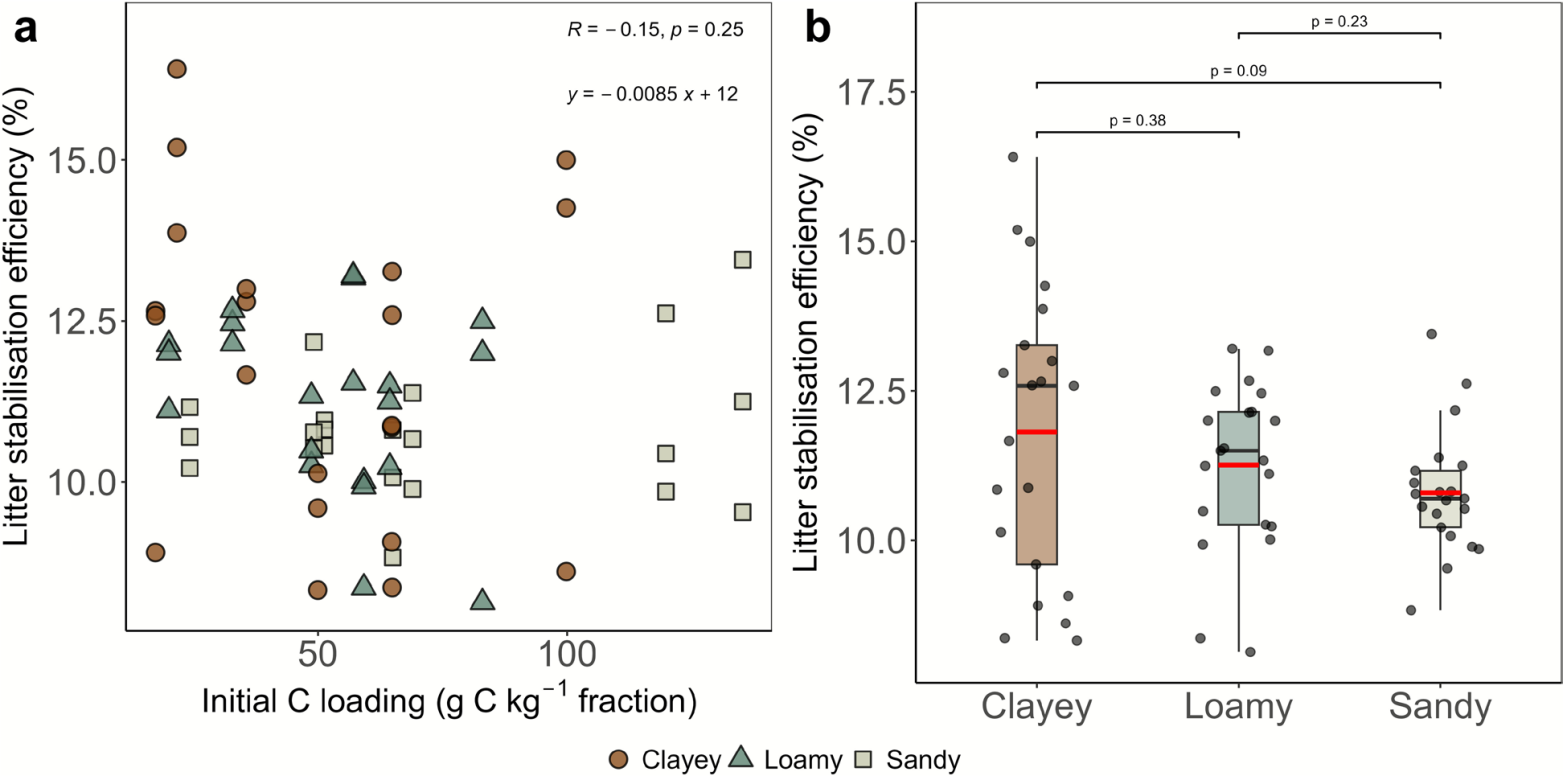
Litter stabilization efficiency (%) in relation to initial OC_fine_ loading of fine fraction (g kg^−1^ fraction) for the three texture classes (a) and comparison of litter stabilization efficiency across clayey, loamy, and sandy soils (b). Red line in boxplots display the mean and black line the median.

Litter stabilization efficiency represents the proportion of added litter that is retained and stabilized as OC_fine_ in relation to the total litter decomposition by accounting for all litter that remained in OC_coarse_. This showed no significant relationship with initial OC loading of the fine fraction (Figure 4a). While there was a slight decrease as initial fine fraction OC loading increased, the overall trend was weak (*p* = 0.5). When comparing across soil textures, clayey soils exhibited the highest variability in litter stabilization efficiency but also the highest mean stabilization efficiency, while loamy and sandy soils showed less variability and lower values (Figure 4b). The litter stabilization efficiency was not different between the clayey and loamy soils nor the loamy and sandy soils. The clayey and sandy soils showed substantial differences in the litter stabilization efficiency, with on average more retained litter‐derived OC_fine_ in the clayey soils.

### 3.4 Microscale Distribution of ^13^C‐Labeled Litter‐Derived SOC (Patches Across the Heterogeneous Organo–Mineral Interactions)

**FIGURE 5:**
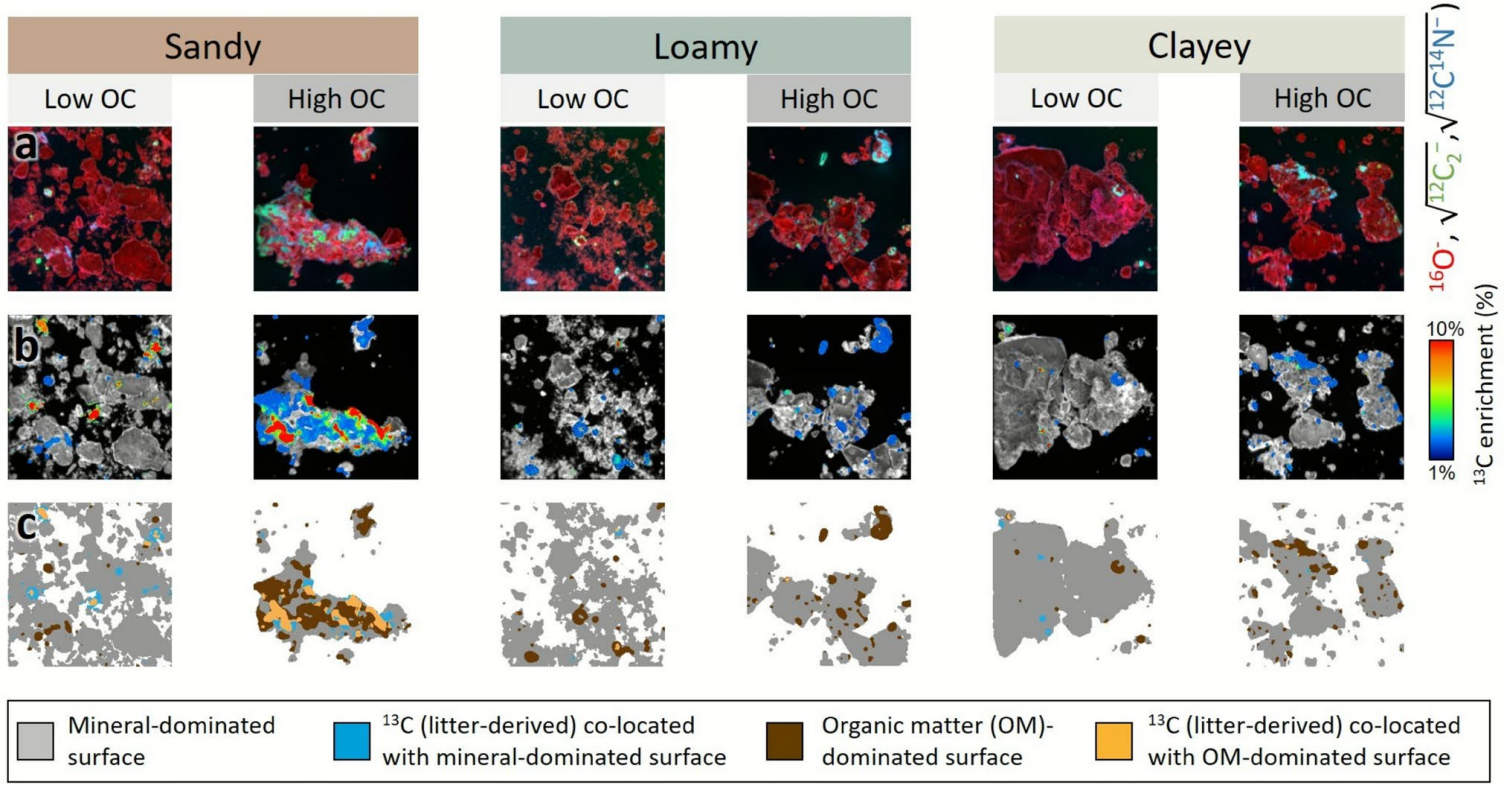
Spatial patterns and co‐location of litter‐derived ^13^C in soils of varying texture and organic carbon (OC) content. (a) The first row of images displays the distribution of O, C, and N in RGB coloring. (b) ^13^C enrichment maps highlighting litter‐derived C hotspots. (c) Machine learning segmentation of mineral, OM, and ^13^C‐enriched areas. Further details on the image analysis are provided in the SI, and all considered images are shown in Figures S4–S6.

**FIGURE 6:**
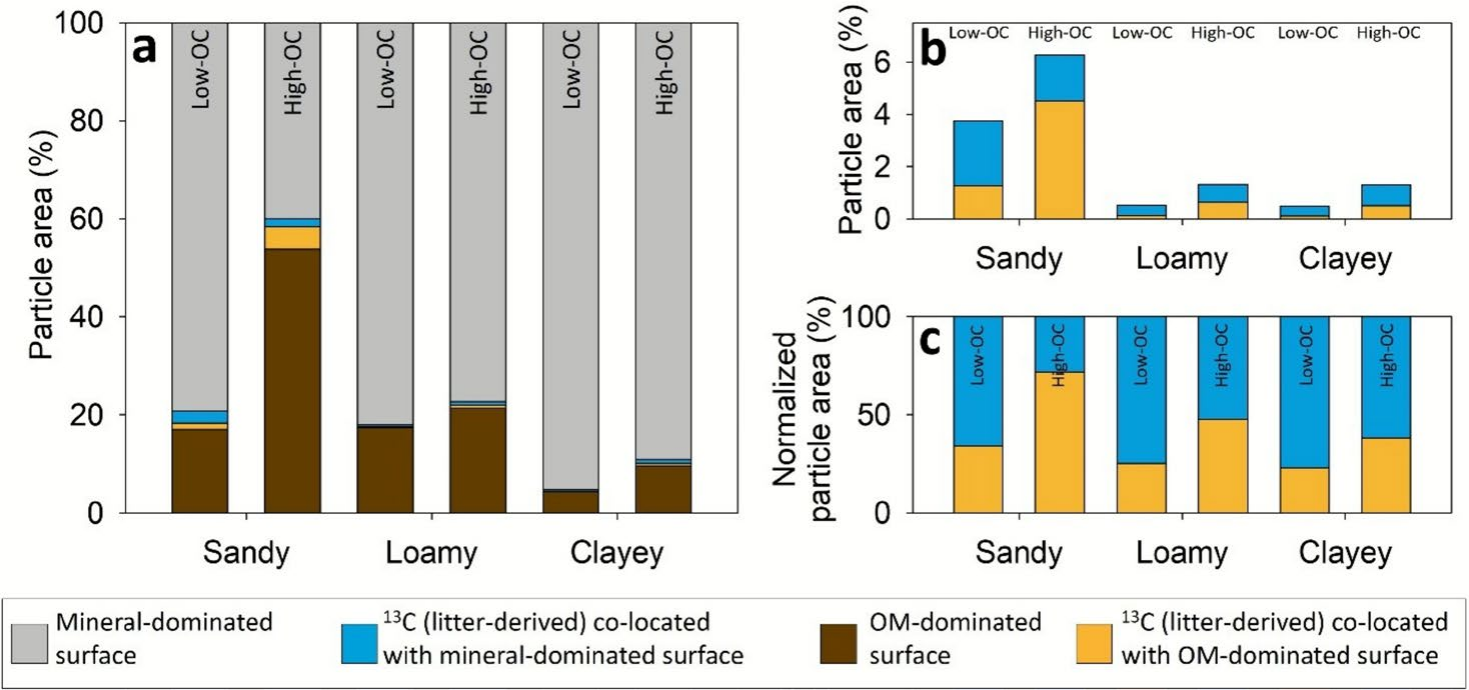
Distribution of organic carbon (OC) across different soil textures and surface types. (a) Proportions of particle area (%) in sandy, loamy, and clayey soils with low and high OC content, highlighting the distribution of mineral‐dominated surface (gray), organic matter (OM)‐dominated surface (orange), and litter‐derived OC co‐localized with mineral (blue) or OM‐dominated (yellow) surfaces. (b) Particle area (%) of litter‐derived OC in different soil types. (c) Normalized particle area (%) distribution of litter‐derived OC and surface interactions across sandy, loamy, and clayey soils.

Across all fine particle content classes, the SOC was found in distinct soil OM patches in the analyzed soil fine fractions (Figure 5a). Based on the segmentation, we estimated the total analyzed area to be pure mineral surface, covered by native OM and derived from labeled litter co‐allocated with mineral surfaces or native OM. Overall, the surface cover with OM was higher in High‐OC soils (Figures 5c and 6a). The mineral‐dominated surfaces, which were free of OM, strongly prevailed in all soils with 77%–95% except for High‐OC sandy soil, which had an OM‐dominated surface coverage of 54% (Figure 6a). Considering the recovered ^13^C, the fraction of litter‐derived OC that was associated with native OM was always higher in high‐OC compared to the low‐OC soils (Figure 6b,c). In the High‐OC sandy soil, ~75% of the litter‐derived OC was co‐localized with already existing OM‐dominated surfaces. Figure S3 shows the ratio of OM‐dominated to mineral‐dominated surface areas (representing the expected distribution of new OC) compared to the actual observed recovery. In all cases, observed recovery on OM‐rich surfaces exceeded the expected values, suggesting a preferential formation of new OC on preexisting OM. Interestingly, in relative terms this difference was particularly pronounced in the clayey soils (Figure 6). So, the soils with the highest surface area, with vast uncovered surface area, showed the strongest tendency of OC formation on preexisting OM patches. Notably, sandy soils showed elevated levels of ^13^C enrichment across both OM and mineral surfaces, whereas loamy and clayey soils displayed comparatively lower litter‐derived ^13^C patches per particle area.

## 4 Discussion

### 4.1 Initial SOC Shapes Litter‐Derived SOC Recovery

After 2 years, about 22% (on average) of the added litter‐derived OC was retained, while around 80% was lost via microbial mineralization. These results are consistent with broader trends reported in the literature, including similar observations by Poeplau et al. (2023) and Gregorich et al. (2023), and reinforce the prevailing understanding that most plant debris that enters the soil is rapidly turned over, with only a small fraction recovered as litter‐derived SOC. The recovery of litter‐derived OC in bulk soils increased with increasing initial SOC content (Figure 3). This increase was primarily driven by the enhanced recovery of litter‐derived OC_coarse_, indicating lower decomposition of added litter in High‐OC soils. Wu et al. (2024) reported similar findings from a short‐term (90‐day) incubation experiment, showing that soils with higher initial SOC retained more added OC and emitted less CO_2_ derived from the new litter, indicating greater microbial C use efficiency. In contrast, C‐depleted soils mineralized a larger share of the fresh input as CO_2_ due to stronger microbial priming, highlighting that initial SOC strongly determines the fate of fresh OM and the efficiency of SOC sequestration (Wu et al. 2024). However, long‐term field studies, such as Sanderman et al. (2017), provide a contrasting perspective showing that High‐OC soils had faster C turnover, driven by greater plant productivity and microbial activity. In our study, litter‐derived OC_coarse_ was significantly higher in High‐OC soils (Figure 3b), suggesting that a substantial portion of the added litter remained undecomposed and was retained in the OC_coarse_. In contrast, the recovery of litter‐derived OC_fine_ declined with increasing SOC (Figure 3c). This indicates that in High‐OC soils, a smaller portion of the added litter was utilized and finally transformed into forms that associate with mineral surfaces, resulting in reduced stabilization in OC_fine_. A likely explanation is that the litter added has a C:N ratio of 56, which is fairly typical for plant residues and reflects common conditions where nitrogen can be limiting. In Low‐OC soils, microbes are strongly C limited, with a fresh source of C being decomposed instantaneously as an important source of energy (Figure 2). However, along this gradient of initial SOC, this might have shifted towards a limitation of nitrogen instead of C (Craig et al. 2021). In High‐OC soils, the turnover of native OM may be higher, leaving the added fresh litter less decomposed. Thus, while microbial growth may be more efficient in High‐OC soils, incorporation of new C into OC_fine_ was reduced, reflecting a shift in microbial processing rather than surface area limitation, but likely due to altered processing pathways. Similar mechanisms have been linked to microbial adjustments in C‐N balance through necromass recycling. Cui et al. (2020) and Kaiser et al. (2014) showed that under high C inputs, microbes recycle nitrogen from necromass rather than decompose native OM. In our case, microbes in High‐OC soils may similarly recycle nitrogen through necromass turnover, thereby reducing the formation of new litter‐derived OC_fine_. Supporting this, Malik et al. (2019), Sinsabaugh et al. (2013), found that in Low‐OC soils, microbes invest more energy to access resources, reducing C use efficiency and increasing CO_2_ loss, while High‐OC soils support more efficient microbial growth and greater C retention, highlighting how SOC content and microbial activity together shape C storage. Finally, physical protection mechanisms may also contribute, as High‐OC soils typically have better aggregation and structure, which can occlude coarse litter particles and limit microbial access (Angst et al. 2021). Together, these factors may explain the persistence of litter‐derived OC_coarse_ with high initial SOC.

### 4.2 Limited Influence of Texture and on the Stabilization Efficiency of Litter Incorporated Into OC_fine_

Interestingly, the stabilization efficiency of litter incorporated into OC_fine_ varied only slightly but not significantly across different initial OC loadings of fine fraction. The initial OC loading of fine fraction was recorded higher in sandy soils (24–135 g C kg^−1^ fine fraction) and lower in clayey soils (17–100 g C kg^−1^ fine fraction), a pattern also reported by Begill et al. (2023). Yet, even at OC loading of fine fraction reaching 135 (g C kg^−1^ fine fraction), the formation of litter‐derived OC_fine_ persisted. Based on global data, it is suggested that 86 g C kg^−1^ fine fraction represents a maximum loading (Georgiou et al. 2022). As we used silt + clay content as a proxy for mineral surface availability, our findings underline that stabilization can occur beyond these proposed limits. However, in High‐OC soils, this did not result in a net increase in litter‐derived OC_fine_ formation (Figure S7). This indicates that mineral surface availability alone does not limit stabilization; thus, additional mechanisms may operate. In particular, aggregation, which is typically enhanced under higher SOC and finer textures, is acknowledged to stabilize OC (Six et al. 2002; Totsche et al. 2018; Angst et al. 2021), but was ignored in this two‐pool fractionation approach.

This study is limited to directly test whether soil texture or OC content influences the soil's capacity to stabilize new OC, since new OC could have simply replaced old OC without being influenced by the availability of mineral surfaces. However, the NanoSIMS images revealed that even in High‐OC soils (sandy, loamy, and clayey soils with 122, 83, and 65 g C kg^−1^ fine fraction), the majority (> 50%) of new litter‐derived OC_fine_ formation was allocated at mineral dominated sites. At these sites, it is less likely that the formation of OC patches triggered the turnover of native OC_fine_, indicating that the formation of new OC_fine_ is not limited by the availability of binding surfaces.

Surprisingly, the stabilization efficiency across all textures was not statistically significant (*p* > 0.05; Table S4), further supporting the idea that available mineral surfaces were not a major limitation for OC_fine_ formation. The measured stabilization efficiency was 11.8% (ranging between 8% and 16%) in clayey soils, 11.2% (8%–13%) in loamy soils, and 10.7% (8%–13%) in sandy soils, showing a slight but nonsignificant modulation by texture. This contrasts with the established understanding that the fate of litter in soils is strongly influenced by clay content (Liu et al. 2014; Coleman and Jenkinson 1996). Higher SOC contents are often observed in clayey soils despite similar C inputs (Poeplau et al. 2021). It is possible that this is related to the incubation set‐up, which is not directly comparable with field conditions. Under field conditions, C entering sandy soils might be mineralized before it interacts with clay particles simply because fine particles are very diluted in sandy soils. In contrast, the incubation experiment involved finely ground straw, which was homogenously incorporated into the soil matrix, allowing decomposition and stabilization to occur more or less at the same spot. In a previous study, in which OM was also thoroughly mixed with mineral soil, we also found only slight differences in OC_fine_ formation between coarse and fine‐textured soils (Begill et al. 2025). Also, the interaction with plant roots, fostering aggregation, was excluded in the incubation jars but might be a relevant factor leading to higher stabilization rates of carbon in fine‐textured soils (Kang et al. 2023; Schiedung et al. 2023). The fact that clayey soils also had the highest variability in litter stabilization efficiency might be related to the more complex structure as compared to sandy soils.

Finally, these findings support a growing recognition that OC_fine_ stabilization is not solely a function of mineralogical capacity, but is intricately linked to the dynamic interplay among inputs, microbes, and environmental factors (Poeplau et al. 2024; Kirschbaum et al. 2020).

### 4.3 Litter‐Derived OC Forms Distinct Microscale Patches Across Mineral and Organic Surfaces in the Fine Fraction

Microscale analysis by NanoSIMS of the fine fraction < 20 μm revealed that litter‐derived OC formed distinct μm‐sized patches that were partially distributed across mineral‐dominated and OM‐dominated surfaces. In most soils, the co‐location of litter‐derived ^13^C with the mineral‐dominated surface prevailed, except in the sandy high‐OC (Figure 5d) (Wilhelm et al. 2022). Here, the majority (54%) of the measured surface was covered by OM and thus, litter‐derived OC was mainly co‐localized with OM‐dominated surfaces, presumably representing preexisting native OM patches. Nevertheless, also in all other soils, there was a preferential formation of new OC_fine_ on preexisting OM in relative terms (as indicated by the ratio of free and OM‐dominated surfaces vs. the ratio of mineral and OM co‐located new OC_fine_, Figure S3 and Figure 6). This observation across all soils underscores the significant role of native OC_fine_ for the formation of new OC_fine_ (Kang et al. 2024). The distinct litter‐derived patches are most likely dominated by microbial residues (Angst et al. 2023) and thus are allocated at or next to microbial hotspots where the added litter was decomposed and transformed, while close to pure mineral surfaces the added litter remained as OC_coarse_. The stronger ^13^C signal on sandy surfaces suggests that litter‐derived OC remains less processed and closer to its original form. In contrast, the weaker signal in loamy and clay soils indicates greater microbial incorporation and mixing with native, unlabeled C. This enhanced processing in fine‐textured soils may explain their slightly higher litter‐derived SOC recovery efficiency observed in Figure 4b. These insights and our multi‐texture analysis after 2 years of incubation emphasize the need for SOC perspectives that account for the heterogeneous microscale dynamics of OC stabilization as influenced by organo‐mineral and organo–organo interactions rather than focusing mainly on mineral surface availability.

## 5 Implications

Understanding the stabilization efficiency of newly added OC in relation to existing SOC content is critical for improving predictions of SOC storage and informing sustainable land management practices. Traditionally, stabilization of newly added OC has been thought to be constrained primarily by the availability of fine mineral surfaces (Hassink 1997; Stewart et al. 2009). However, here we show that new OC can be stabilized in the fine fraction in soils with very high initial OC loadings. Even at fine fraction OC loadings of up to 135 g C kg^−1^ fine fraction, newly added litter‐derived OC_fine_ continues to form at a similar rate as in soils with much lower fine fraction OC loadings (Figure 4a). This suggests that stabilization is possible beyond proposed OC loading limits (Georgiou et al. 2022), although we note that our study used silt + clay content as a proxy for mineral surface availability and did not directly measure mineral surface area.

A small proportion of the added litter remained undecomposed as OC_coarse_ after 2 years, with lower losses in High‐OC soils (Figure 2). This indicates that SOC turnover can be triggered by C‐limited conditions. At the same time, native SOC losses increased with increasing SOC content. This pattern suggests that microbes may have preferentially used the fresh material in rather C and energy‐limited Low‐OC soils, while they were not relying on exogenous C of lower quality when initial SOC was high. It might also be a mere stochastic effect, that exoenzymes broke down more old and less new OC in the High‐OC soils, just because microbes were already surrounded by more OM. In either case, the results of this study suggest that the decomposition and fate of OC added to a soil are not independent of preexisting OC, which is the common assumption in first‐order kinetics models. In most classical SOC turnover models, the decomposition of freshly added OC is completely independent of the preexisting SOC (Parton 1996; Parshotam 1996). There had been attempts to formulate saturation or priming effects in such models (Wutzler and Reichstein 2013; Ahrens et al. 2020; Bernard et al. 2022), while the debate is ongoing if either of those two can significantly help to improve simulations of OC dynamics. Here, we potentially identified a novel interactive effect of preexisting SOC and the decay of freshly added OC, which needs to be better understood from a mechanistic point of view. Afterall, this study suggests that at least after 2 years, initial SOC is not limiting the retention of new OC but tends to increase its residence time in the soil.

## Author Contributions

**Neha Begill:** data curation, formal analysis, investigation, visualization, writing – original draft, writing – review and editing. **Steffen A. Schweizer:** data curation, methodology, visualization, writing – review and editing. **Axel Don:** conceptualization, writing – review and editing. **Carmen Hoeschen:** visualization, writing – review and editing. **Marcus Schiedung:** validation, writing – review and editing. **Georg Guggenberger:** writing – review and editing. **Christopher Poeplau:** conceptualization, methodology, supervision, writing – review and editing.

## Funding

This study has received funding from the European Unions' Horizon 2020 research and innovation programme under grant agreement No. 862695 EJP SOIL.

## Conflicts of Interest

The authors declare no conflicts of interest.

## Supporting information


**Appendix S1:** gcb70646‐sup‐0001‐AppendixS1.docx.

## Data Availability

All the data are available in Supporting Information. Additionally, the data supporting this manuscript are available at https://doi.org/10.5281/zenodo.17515877.
