# Supplementary material for: Increased Retention of Litter‐Derived Organic Carbon With Increasing Initial Carbon Content in Temperate Agricultural Soils
Source: Glob Chang Biol. 2025 Dec 8;31(12):e70646. doi: 10.1111/gcb.70646 (PMC12683473; doi:10.1111/gcb.70646)
Supplement: Supplementary file 1 — Appendix S1: gcb70646‐sup‐0001‐AppendixS1.docx. [file GCB-31-e70646-s001.docx]

**Supplementary Materials for**

**Initial carbon content, rather than available mineral surfaces, governs the formation of mineral-associated soil organic carbon**

Neha Begill *et al.*

Thünen Institute of Climate-Smart Agriculture, Bundesallee 68, 38116 Braunschweig, Germany

Corresponding author: [christopher.poeplau@thuenen.de](mailto:axel.don@thuenen.de)

**Supplementary Tables**

**Table S1**. General Parameters of Incubated Soil Samples: Overview of key soil properties, including texture composition (sand, silt, and clay) (%), total organic carbon (TOC) (g kg^-1^), total nitrogen (TN) (g kg^-1^), carbon-to-nitrogen ratio (C/N), land use, pH, and electrical conductivity (EC).

| Texture | Sand | Silt | Clay | TOC | TN | C/N | Landuse | pH | EC |
| --- | --- | --- | --- | --- | --- | --- | --- | --- | --- |
| sandy | 66.7 | 23.1 | 10 | 50.4 | 3.8 | 13.1 | Grassland | 5.6 | 78.8 |
| sandy | 67.7 | 22.1 | 10 | 46.3 | 2.6 | 17.8 | Cropland | 6.6 | 104.6 |
| sandy | 64 | 24.6 | 11 | 31.7 | 2.7 | 11.7 | Special permanent crops | 5.2 | 50 |
| sandy | 67 | 22.8 | 10 | 28 | 2.7 | 10.4 | Grassland | 6.4 | 78.3 |
| sandy | 67.9 | 20.8 | 11 | 22.2 | 2 | 11.3 | Grassland | 5.9 | 77.6 |
| sandy | 64.6 | 24.5 | 11 | 17.3 | 1.5 | 11.2 | Cropland | 6.7 | 197.8 |
| sandy | 66.6 | 23.1 | 10 | 7.1 | 0.6 | 11.8 | Cropland | 6.9 | 79.1 |
| loamy | 46.5 | 31.1 | 22 | 61.2 | 5.2 | 11.8 | Grassland | 5.5 | 43.4 |
| loamy | 43.8 | 33.1 | 23 | 53.5 | 4.2 | 12.6 | Grassland | 5.8 | 335 |
| loamy | 42.6 | 33.8 | 24 | 43 | 4.6 | 9.4 | Grassland | 5.5 | 71.8 |
| loamy | 46.1 | 30.9 | 23 | 37.5 | 3.4 | 10.9 | Grassland | 6.1 | 81.6 |
| loamy | 43.1 | 32.4 | 25 | 24.5 | 2.2 | 11 | Cropland | 6.4 | 100.2 |
| loamy | 48.6 | 31 | 20 | 15.4 | 1.5 | 10.5 | Cropland | 6.8 | 102.8 |
| loamy | 44.6 | 34 | 21 | 9.9 | 1.1 | 8.7 | Cropland | 7.1 | 207 |
| clayey | 8.1 | 49.2 | 43 | 102.2 | 9.7 | 10.6 | Grassland | 5.7 | 175.8 |
| clayey | 9.8 | 41.2 | 49 | 81.2 | 7.3 | 11.2 | Grassland | 6.2 | 161.5 |
| clayey | 12.3 | 45 | 43 | 77.1 | 7.7 | 10 | Grassland | 5.4 | 196.8 |
| clayey | 12.7 | 43.6 | 44 | 53.5 | 5.5 | 9.7 | Grassland | 5.4 | 67.2 |
| clayey | 10.8 | 48.2 | 41 | 31.7 | 3.2 | 9.8 | Special permanent crops | 4.9 | 85.6 |
| clayey | \| 14.7 \| \| --- \| | 41.9 | 43 | 23 | 2.2 | 10.4 | Cropland | 6.6 | 146 |
| clayey | 15.5 | 41.6 | 43 | 16 | 1.7 | 9.7 | Cropland | 6.5 | 45.9 |

***Table S2.*** *NanoSIMS-selected samples across different soil textures (sandy, loamy, and clayey) under litter addition and control conditions, showing bulk SOC (%) and OC loading (%).*

| **S.No** | **Texture** | **Treatment/Control** | **Bulk SOC (%)** | **OC loading (%)** |
| --- | --- | --- | --- | --- |
| 1 | Sandy | treatment | 5.04 | 12.22 |
| 2 | Sandy | Control | 5.04 | 12.46 |
| 3 | Sandy | treatment | 0.71 | 2.66 |
| 4 | Sandy | Control | 0.71 | 2.14 |
| 5 | Loamy | treatment | 6.12 | 8.20 |
| 6 | Loamy | Control | 6.12 | 8.27 |
| 7 | Loamy | treatment | 0.99 | 2.10 |
| 8 | Loamy | Control | 0.99 | 1.83 |
| 9 | Clayey | treatment | 8.12 | 6.49 |
| 10 | Clayey | Control | 8.12 | 6.55 |
| 11 | Clayey | treatment | 1.60 | 1.65 |
| 12 | Clayey | Control | 1.60 | 1.57 |

***Table S3.*** *Raw data for total organic carbon (TOC), texture, δ¹³C, atom% ¹³C, and carbon contents of bulk soil, coarse fraction, and fine fraction across treatments.*

| **Treatment** | **TOC_initial** | **texture** | **δ13C_Bulk (‰)**   \|  \| \| --- \| \|  \| | **Atom%_13C_Bulk** | **C_Bulk (%)** | **δ13C_Coarse (‰)** | **Atom%_13C_Coarse** | **C_Coarse (%)** | **δ13C_Fine (‰)** | **Atom%_13C_Fine** | **C_Fine (%)** |
| --- | --- | --- | --- | --- | --- | --- | --- | --- | --- | --- | --- | --- | --- |
| Control | 50 | Sandy | -27.67 | 1.08 | 4.78 | -28.66 | 1.07 | 2.23 | -28.09 | 1.07 | 12.5 |
| Control | 46 | Sandy | -26.16 | 1.08 | 4.26 | -26.80 | 1.08 | 1.69 | -27.54 | 1.08 | 13.6 |
| Control | 32 | Sandy | -26.60 | 1.08 | 2.98 | -28.59 | 1.07 | 2.02 | -27.64 | 1.08 | 6.1 |
| Control | 28 | Sandy | -28.13 | 1.07 | 2.43 | -29.53 | 1.07 | 1.02 | -28.44 | 1.07 | 6.3 |
| Control | 22 | Sandy | -27.67 | 1.08 | 1.96 | -29.56 | 1.07 | 0.78 | -28.00 | 1.08 | 4.7 |
| Control | 17 | Sandy | -26.43 | 1.08 | 1.60 | -27.69 | 1.08 | 0.44 | -27.37 | 1.08 | 4.9 |
| Control | 7 | Sandy | -26.14 | 1.08 | 0.61 | -28.39 | 1.07 | 0.13 | -26.86 | 1.08 | 2.1 |
| treatment_1 | 50 | Sandy | 171.32 | 1.29 | 4.68 | 274.67 | 1.41 | 2.26 | 145.45 | 1.26 | 12.2 |
| treatment_2 | 50 | Sandy | 164.92 | 1.29 | 3.67 | 376.35 | 1.52 | 2.44 | 135.26 | 1.25 | 11.8 |
| treatment_3 | 50 | Sandy | 237.77 | 1.36 | 4.83 | 378.96 | 1.52 | 2.50 | 147.75 | 1.27 | 12.2 |
| treatment_1 | 46 | Sandy | 181.37 | 1.30 | 4.21 | 508.27 | 1.66 | 1.68 | 136.46 | 1.25 | 13.1 |
| treatment_2 | 46 | Sandy | 192.15 | 1.32 | 3.88 | 537.22 | 1.69 | 2.02 | 148.62 | 1.27 | 12.9 |
| treatment_3 | 46 | Sandy | 195.58 | 1.32 | 4.57 | 533.94 | 1.69 | 1.84 | 124.79 | 1.24 | 13.1 |
| treatment_1 | 32 | Sandy | 325.00 | 1.46 | 2.38 | 992.10 | 2.18 | 1.09 | 252.31 | 1.38 | 6.2 |
| treatment_2 | 32 | Sandy | 351.26 | 1.49 | 2.31 | 750.33 | 1.92 | 0.96 | 248.36 | 1.38 | 6.1 |
| treatment_3 | 32 | Sandy | 338.66 | 1.47 | 2.51 | 738.03 | 1.91 | 1.14 | 237.87 | 1.37 | 6.2 |
| treatment_1 | 28 | Sandy | 327.55 | 1.46 | 2.66 | 563.66 | 1.72 | 1.05 | 333.32 | 1.47 | 6.7 |
| treatment_2 | 28 | Sandy | 401.21 | 1.54 | 2.48 | 651.24 | 1.81 | 1.13 | 291.71 | 1.42 | 6.6 |
| treatment_3 | 28 | Sandy | 368.23 | 1.51 | 2.45 | 477.02 | 1.62 | 1.05 | 284.36 | 1.42 | 6.5 |
| treatment_1 | 22 | Sandy | 454.53 | 1.60 | 1.74 | 631.16 | 1.79 | 0.77 | 419.32 | 1.56 | 4.9 |
| treatment_2 | 22 | Sandy | 752.38 | 1.92 | 1.97 | 951.22 | 2.13 | 0.89 | 404.34 | 1.55 | 4.8 |
| treatment_3 | 22 | Sandy | 562.31 | 1.72 | 1.81 | 779.77 | 1.95 | 0.80 | 394.85 | 1.54 | 4.7 |
| treatment_1 | 17 | Sandy | 589.34 | 1.75 | 1.56 | 1104.58 | 2.30 | 0.49 | 420.72 | 1.56 | 5.1 |
| treatment_2 | 17 | Sandy | 530.60 | 1.68 | 1.62 | 812.26 | 1.99 | 0.42 | 431.26 | 1.57 | 4.9 |
| treatment_3 | 17 | Sandy | 647.77 | 1.81 | 1.69 | 1177.14 | 2.38 | 0.54 | 368.03 | 1.51 | 5.1 |
| treatment_1 | 7 | Sandy | 1340.04 | 2.55 | 0.73 | 2109.48 | 3.36 | 0.13 | 1018.19 | 2.21 | 2.7 |
| treatment_2 | 7 | Sandy | 1335.75 | 2.54 | 0.74 | 2171.20 | 3.42 | 0.14 | 1032.54 | 2.22 | 2.7 |
| treatment_3 | 7 | Sandy | 1318.43 | 2.53 | 0.67 | 2228.56 | 3.48 | 0.11 | 1021.59 | 2.21 | 2.6 |
| Control | 61 | Loamy | -26.93 | 1.08 | 5.19 | -27.93 | 1.08 | 3.80 | -26.90 | 1.08 | 8.3 |
| Control | 54 | Loamy | -27.52 | 1.08 | 4.52 | -28.38 | 1.07 | 3.05 | -28.00 | 1.08 | 6.1 |
| Control | 43 | Loamy | -27.28 | 1.08 | 3.33 | -28.44 | 1.07 | 1.76 | -27.62 | 1.08 | 6.1 |
| Control | 38 | Loamy | -27.59 | 1.08 | 3.12 | -29.76 | 1.07 | 1.41 | -28.27 | 1.07 | 5.6 |
| Control | 25 | Loamy | -26.42 | 1.08 | 2.50 | -28.36 | 1.07 | 0.47 | -27.43 | 1.08 | 4.8 |
| Control | 15 | Loamy | -26.75 | 1.08 | 1.44 | -28.47 | 1.07 | 0.31 | -27.30 | 1.08 | 3.2 |
| Control | 10 | Loamy | -20.89 | 1.08 | 0.92 | -27.49 | 1.08 | 0.29 | -25.81 | 1.08 | 1.8 |
| treatment_1 | 61 | Loamy | 161.98 | 1.28 | 5.01 | 195.40 | 1.32 | 2.71 | 127.24 | 1.24 | 8.2 |
| treatment_2 | 61 | Loamy | 153.25 | 1.27 | 4.55 | 186.34 | 1.31 | 4.71 | 132.70 | 1.25 | 8.2 |
| treatment_3 | 61 | Loamy | 149.23 | 1.27 | 4.53 | 118.58 | 1.24 | 4.16 | 140.78 | 1.26 | 8.1 |
| treatment_1 | 54 | Loamy | 253.85 | 1.38 | 4.60 | 347.99 | 1.48 | 4.44 | 156.38 | 1.28 | 6.1 |
| treatment_2 | 54 | Loamy | 308.98 | 1.44 | 4.92 | 160.02 | 1.28 | 5.43 | 178.47 | 1.30 | 5.5 |
| treatment_3 | 54 | Loamy | 134.75 | 1.25 | 4.41 | 164.67 | 1.29 | 4.79 | 194.59 | 1.32 | 5.6 |
| treatment_1 | 43 | Loamy | 211.14 | 1.34 | 3.95 | 811.13 | 1.98 | 2.23 | 122.39 | 1.24 | 6.2 |
| treatment_2 | 43 | Loamy | 228.70 | 1.36 | 3.97 | 688.39 | 1.85 | 2.50 | 136.62 | 1.25 | 6.2 |
| treatment_3 | 43 | Loamy | 238.53 | 1.37 | 4.08 | 860.45 | 2.04 | 2.16 | 154.39 | 1.27 | 6.3 |
| treatment_1 | 38 | Loamy | 248.28 | 1.38 | 3.48 | 540.78 | 1.69 | 1.88 | 176.61 | 1.30 | 5.6 |
| treatment_2 | 38 | Loamy | 280.46 | 1.41 | 3.48 | 573.44 | 1.73 | 1.11 | 202.41 | 1.33 | 5.6 |
| treatment_3 | 38 | Loamy | 265.95 | 1.40 | 3.50 | 648.12 | 1.81 | 1.70 | 198.45 | 1.32 | 5.6 |
| treatment_1 | 25 | Loamy | 371.75 | 1.51 | 2.38 | 1524.12 | 2.74 | 0.62 | 232.20 | 1.36 | 4.9 |
| treatment_2 | 25 | Loamy | 318.68 | 1.45 | 2.38 | 1370.85 | 2.58 | 0.68 | 221.49 | 1.35 | 5.0 |
| treatment_3 | 25 | Loamy | 422.98 | 1.57 | 2.44 | 1121.60 | 2.32 | 0.64 | 234.93 | 1.36 | 4.8 |
| treatment_1 | 15 | Loamy | 717.11 | 1.88 | 1.56 | 1807.29 | 3.04 | 0.37 | 480.60 | 1.63 | 3.4 |
| treatment_2 | 15 | Loamy | 752.72 | 1.92 | 1.57 | 1383.58 | 2.60 | 0.48 | 486.24 | 1.63 | 3.3 |
| treatment_3 | 15 | Loamy | 651.52 | 1.81 | 1.54 | 1791.03 | 3.03 | 0.39 | 481.79 | 1.63 | 3.5 |
| treatment_1 | 10 | Loamy | 1019.45 | 2.21 | 1.02 | 1825.53 | 3.06 | 0.29 | 824.94 | 2.00 | 2.1 |
| treatment_2 | 10 | Loamy | 846.55 | 2.02 | 1.01 | 1457.71 | 2.67 | 0.30 | 743.36 | 1.91 | 2.1 |
| treatment_3 | 10 | Loamy | 930.08 | 2.11 | 1.06 | 1677.00 | 2.91 | 0.27 | 819.81 | 1.99 | 2.1 |
| Control | 102 | Clayey | -28.52 | 1.07 | 8.80 | -29.16 | 1.07 | 7.80 | -28.70 | 1.07 | 9.2 |
| Control | 81 | Clayey | -27.73 | 1.08 | 6.94 | -28.77 | 1.07 | 8.68 | -27.96 | 1.08 | 6.5 |
| Control | 77 | Clayey | -28.33 | 1.07 | 6.82 | -29.17 | 1.07 | 7.06 | -28.67 | 1.07 | 6.4 |
| Control | 53 | Clayey | -27.71 | 1.08 | 4.52 | -29.19 | 1.07 | 4.21 | -27.96 | 1.08 | 4.5 |
| Control | 32 | Clayey | -26.80 | 1.08 | 2.48 | -29.02 | 1.07 | 1.15 | -27.72 | 1.08 | 3.4 |
| Control | 23 | Clayey | -26.80 | 1.08 | 1.92 | -26.46 | 1.08 | 2.01 | -25.64 | 1.08 | 2.0 |
| Control | 16 | Clayey | -24.77 | 1.08 | 1.57 | -27.90 | 1.08 | 1.03 | -26.55 | 1.08 | 1.6 |
| treatment_1 | 102 | Clayey | 179.31 | 1.30 | 8.68 | 167.45 | 1.29 | 4.64 | 66.98 | 1.18 | 8.9 |
| treatment_2 | 102 | Clayey | 83.01 | 1.20 | 8.44 | 249.21 | 1.38 | 9.44 | 67.55 | 1.18 | 9.4 |
| treatment_3 | 102 | Clayey | 176.91 | 1.30 | 8.79 | 147.94 | 1.27 | 9.08 | 84.45 | 1.20 | 9.6 |
| treatment_1 | 81 | Clayey | 221.58 | 1.35 | 7.30 | 445.49 | 1.59 | 10.85 | 69.44 | 1.18 | 6.5 |
| treatment_2 | 81 | Clayey | 136.21 | 1.25 | 7.25 | 205.20 | 1.33 | 10.24 | 60.23 | 1.17 | 6.3 |
| treatment_3 | 81 | Clayey | 145.73 | 1.26 | 7.39 | 378.43 | 1.52 | 10.55 | 78.29 | 1.19 | 6.6 |
| treatment_1 | 77 | Clayey | 185.37 | 1.31 | 6.57 | 303.01 | 1.44 | 7.41 | 87.67 | 1.20 | 6.7 |
| treatment_2 | 77 | Clayey | 180.74 | 1.30 | 7.00 | 393.60 | 1.53 | 7.87 | 88.28 | 1.20 | 6.4 |
| treatment_3 | 77 | Clayey | 170.97 | 1.29 | 6.58 | 173.37 | 1.29 | 6.76 | 82.10 | 1.20 | 6.6 |
| treatment_1 | 53 | Clayey | 127.03 | 1.24 | 4.68 | 276.28 | 1.41 | 4.14 | 137.11 | 1.26 | 4.5 |
| treatment_2 | 53 | Clayey | 147.64 | 1.27 | 4.86 | 460.29 | 1.61 | 4.80 | 121.04 | 1.24 | 4.6 |
| treatment_3 | 53 | Clayey | 145.39 | 1.26 | 4.74 | 408.24 | 1.55 | 3.19 | 120.26 | 1.24 | 4.5 |
| treatment_1 | 32 | Clayey | 214.81 | 1.34 | 3.18 | 344.39 | 1.48 | 1.96 | 276.17 | 1.41 | 3.4 |
| treatment_2 | 32 | Clayey | 319.79 | 1.45 | 3.50 | 495.16 | 1.64 | 1.95 | 259.36 | 1.39 | 3.4 |
| treatment_3 | 32 | Clayey | 251.06 | 1.38 | 3.21 | 556.50 | 1.71 | 2.00 | 263.26 | 1.39 | 3.5 |
| treatment_1 | 23 | Clayey | 658.73 | 1.82 | 2.24 | 859.46 | 2.04 | 2.79 | 420.64 | 1.56 | 2.0 |
| treatment_2 | 23 | Clayey | 560.88 | 1.72 | 2.18 | 1058.07 | 2.25 | 2.62 | 414.67 | 1.56 | 2.1 |
| treatment_3 | 23 | Clayey | 549.06 | 1.70 | 2.16 | 702.04 | 1.87 | 2.30 | 476.22 | 1.62 | 2.1 |
| treatment_1 | 16 | Clayey | 630.94 | 1.79 | 1.65 | 1196.94 | 2.40 | 1.20 | 579.64 | 1.74 | 1.6 |
| treatment_2 | 16 | Clayey | 668.30 | 1.83 | 1.64 | 1119.54 | 2.31 | 1.08 | 537.10 | 1.69 | 1.7 |
| treatment_3 | 16 | Clayey | 708.05 | 1.87 | 1.62 | 892.37 | 2.07 | 1.22 | 538.99 | 1.69 | 1.6 |

***Supplementary Table S4.*** *Results of two-way ANOVA testing the effects of soil texture and initial OC loading on litter stabilization efficiency.*

| **Source of variation** | **df** | **Sum Sq** | **Mean Sq** | **F value** | **p value** |
| --- | --- | --- | --- | --- | --- |
| Texture | 2 | 10.85 | 5.424 | 1.81 | 0.174 |
| Initial OC loading | 1 | 1.28 | 1.276 | 0.43 | 0.517 |
| Texture × Initial OC loading | 2 | 7.10 | 3.549 | 1.18 | 0.314 |
| Residuals | 57 | 171.33 | 3.006 |  |  |

**Supplementary Figures**


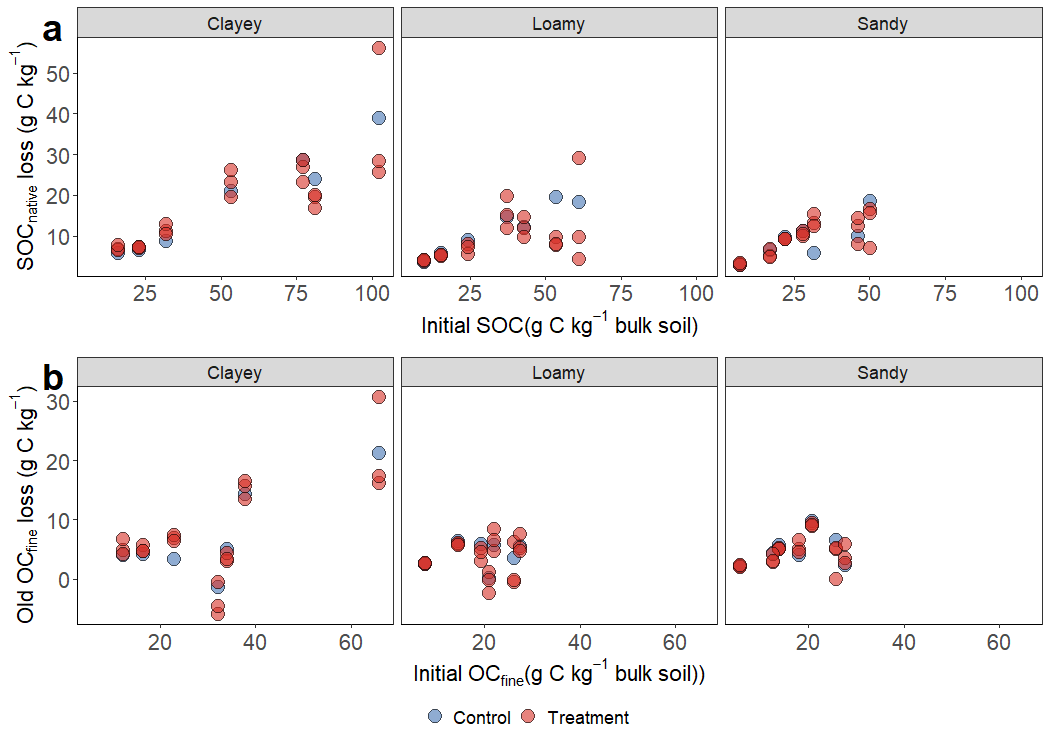


**Supplementary Figure S1:** Relationship between initial soil organic carbon (SOC) content and absolute losses of native SOC(a); and initial fine fraction carbon (OC_fine_) and absolute native OC_fine_ losses (b) across soil textures under control and treatment conditions.


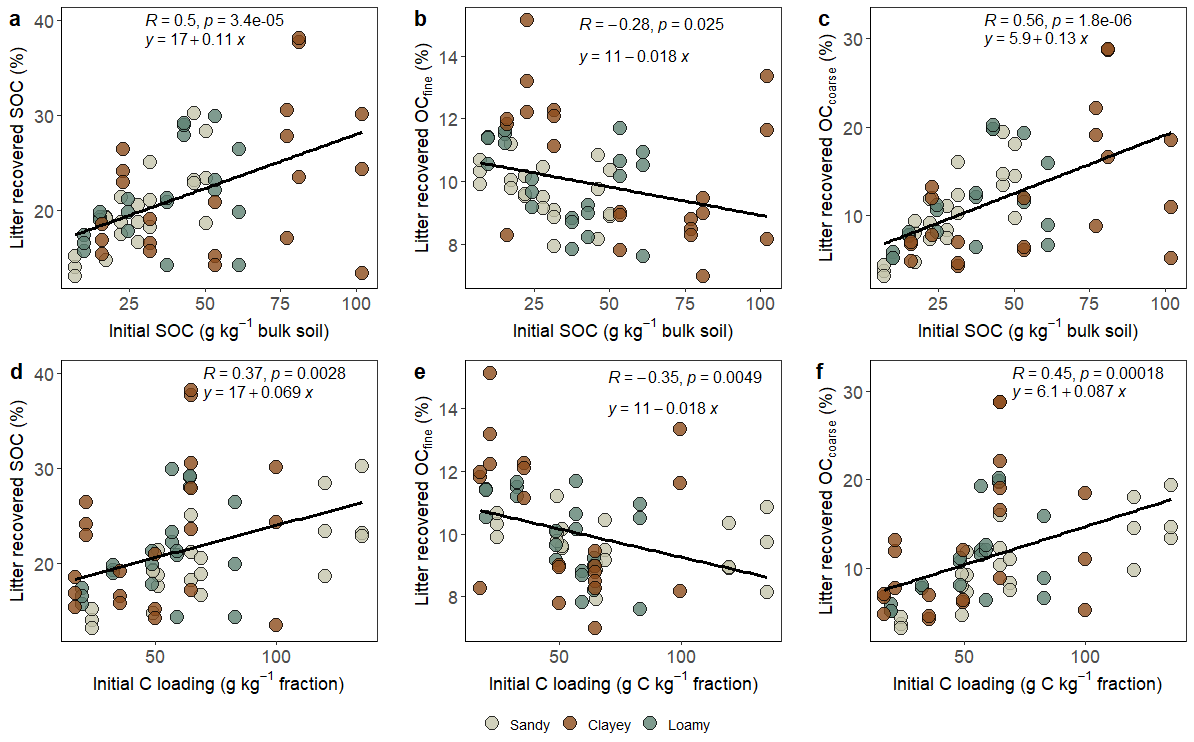


***Supplementary Figure S2****: Relationships between recovered litter-derived organic carbon (OC) in bulk soil, coarse fraction organic carbon (OC_coarse_), and fine fraction organic carbon (OC_fine_) with initial soil organic carbon (SOC) (g C kg^-1^ bulk soil) (a–c) and organic carbon (OC) loading (g OC kg^-1^ silt+clay) of fine fraction (d–f) across different soil textures classes (clayey, loamy, sandy).*

**
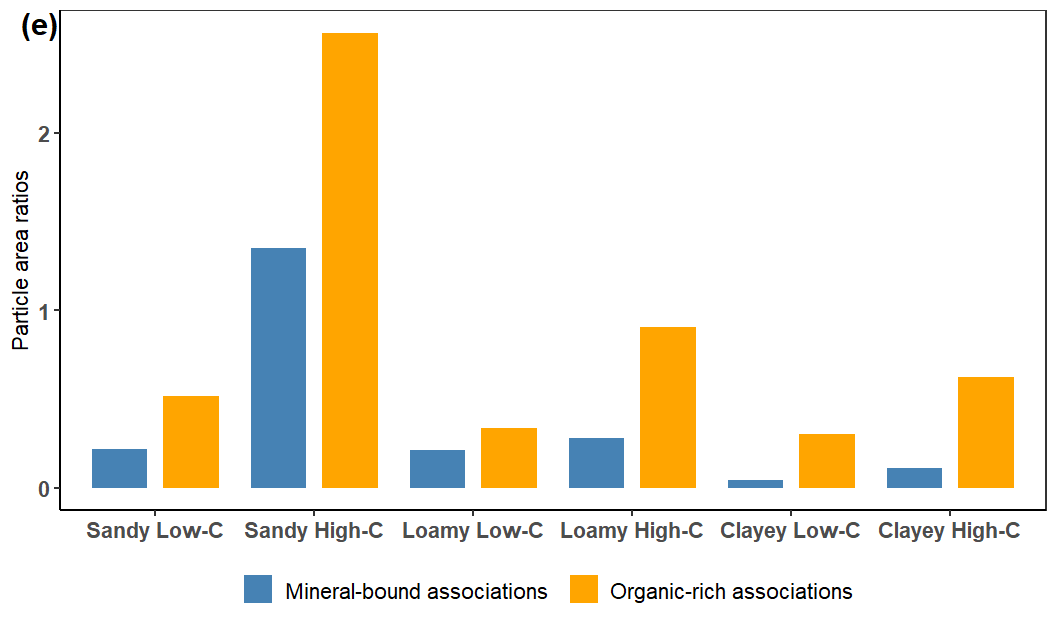
**

***Supplementary Figure S3:*** *Particle area ratios of mineral-bound and organic-rich associations across soils differing in texture and carbon content.*

**NanoSIMS raw data:**


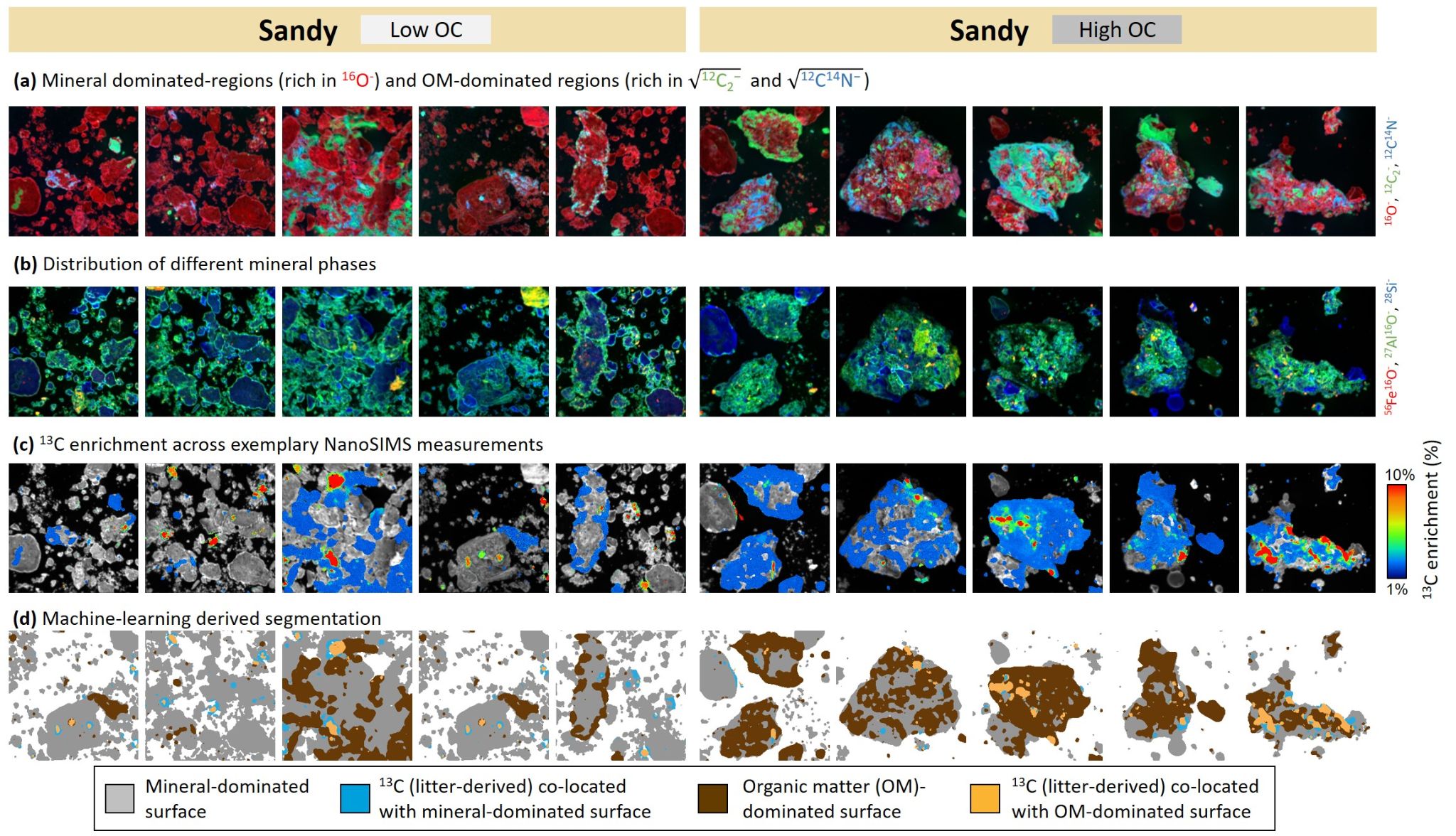


**Supplementary Figure S4**: Spatial distribution and isotopic enrichment patterns of litter-derived carbon (¹³C) across **sandy soil** particles with contrasting organic carbon (OC) content (a), the first row of images displays the distribution of O, C, and N in RGB coloring. (b) Distribution of different mineral phases (c) ¹³C enrichment maps showing spatial incorporation of litter-derived ¹³C across exemplary particles, with higher enrichment visible in high OC samples (d) Machine learning-based segmentation of particle surfaces into mineral-dominated (grey), OM-dominated (brown), and ¹³C-labeled litter-derived regions co-located with either mineral (blue) or OM (yellow) surfaces.


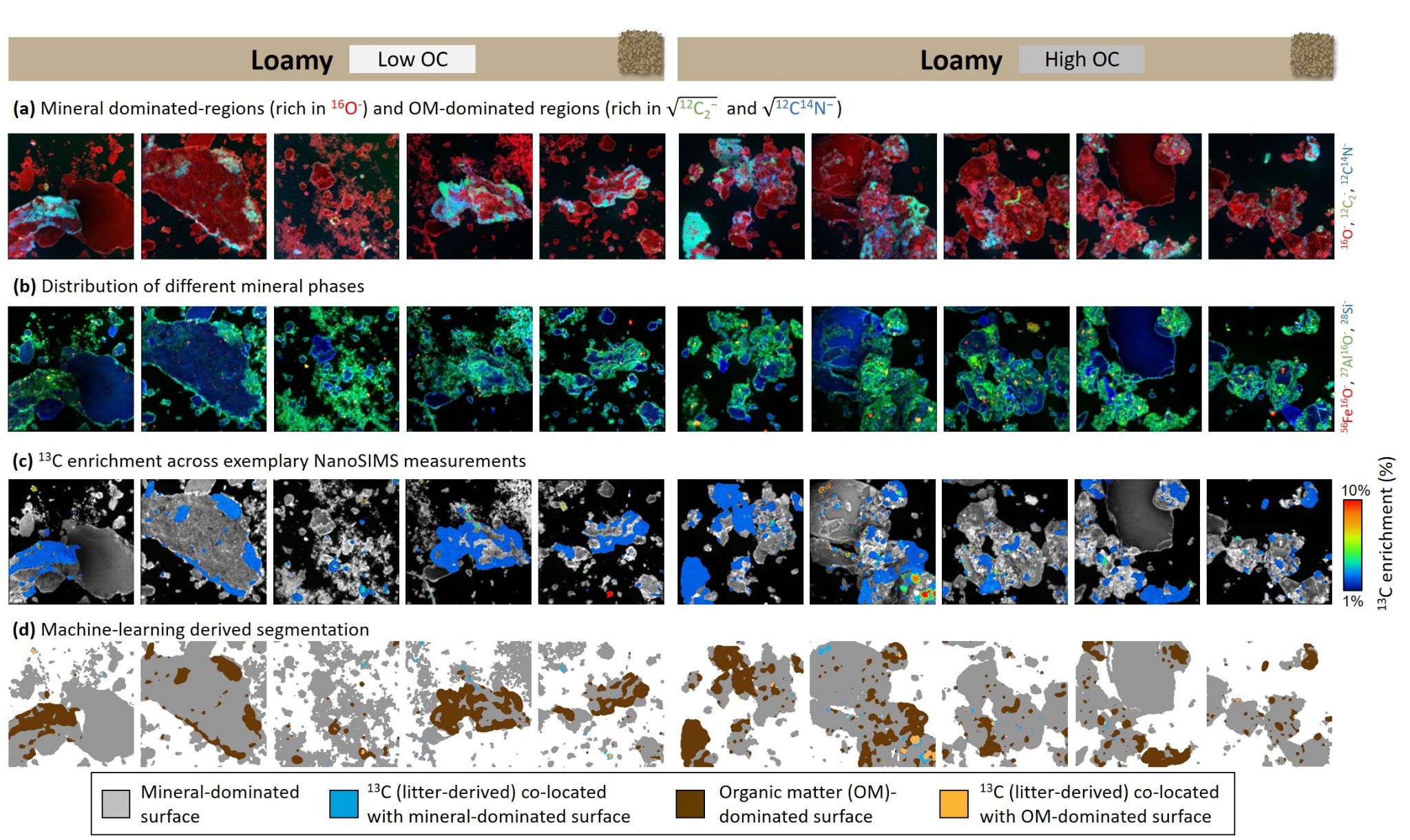


**Supplementary Figure S5**: Spatial distribution and isotopic enrichment patterns of litter-derived carbon (¹³C) across **Loamy soil** particles with contrasting organic carbon (OC) content (a), the first row of images displays the distribution of O, C, and N in RGB coloring. (b) Distribution of different mineral phases (c) ¹³C enrichment maps showing spatial incorporation of litter-derived ¹³C across exemplary particles, with higher enrichment visible in high OC samples (d) Machine learning-based segmentation of particle surfaces into mineral-dominated (grey), OM-dominated (brown), and ¹³C-labeled litter-derived regions co-located with either mineral (blue) or OM (yellow) surfaces.


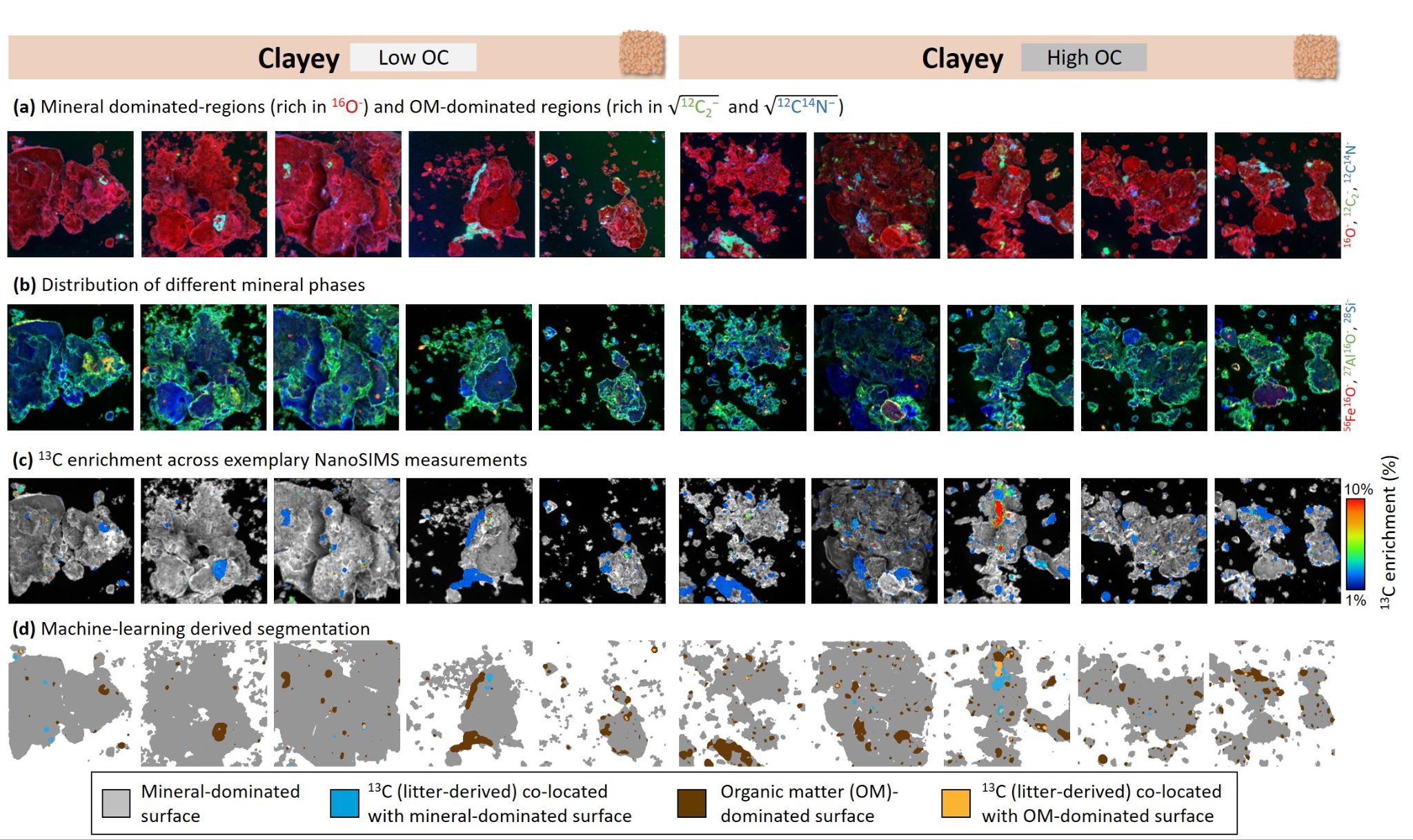


**Supplementary Figure S6**: Spatial distribution and isotopic enrichment patterns of litter-derived carbon (¹³C) across **Clayey** soil particles with contrasting organic carbon (OC) content (a), the first row of images displays the distribution of O, C, and N in RGB coloring. (b) Distribution of different mineral phases (c) ¹³C enrichment maps showing spatial incorporation of litter-derived ¹³C across exemplary particles, with higher enrichment visible in high OC samples (d) Machine learning-based segmentation of particle surfaces into mineral-dominated (grey), OM-dominated (brown), and ¹³C-labeled litter-derived regions co-located with either mineral (blue) or OM (yellow) surfaces.

**
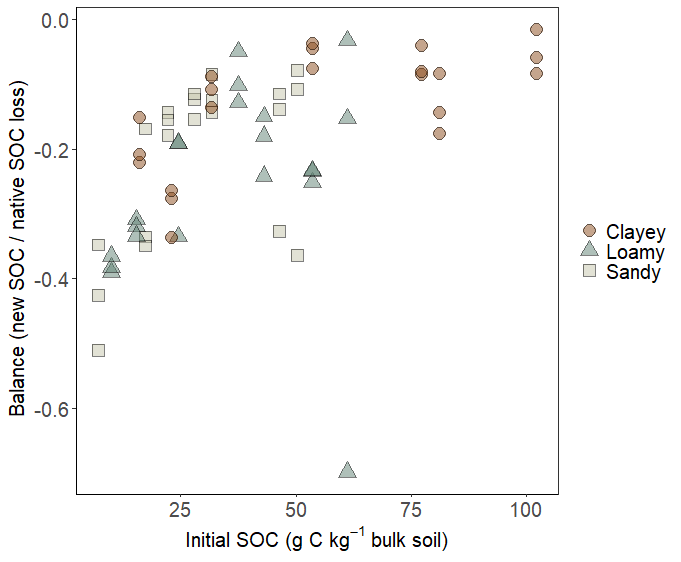
**

**Supplementary Figure S7.** *Balance of new SOC formation relative to native SOC loss as a function of initial SOC. Values near 0 show better compensation; negative values show net loss.*


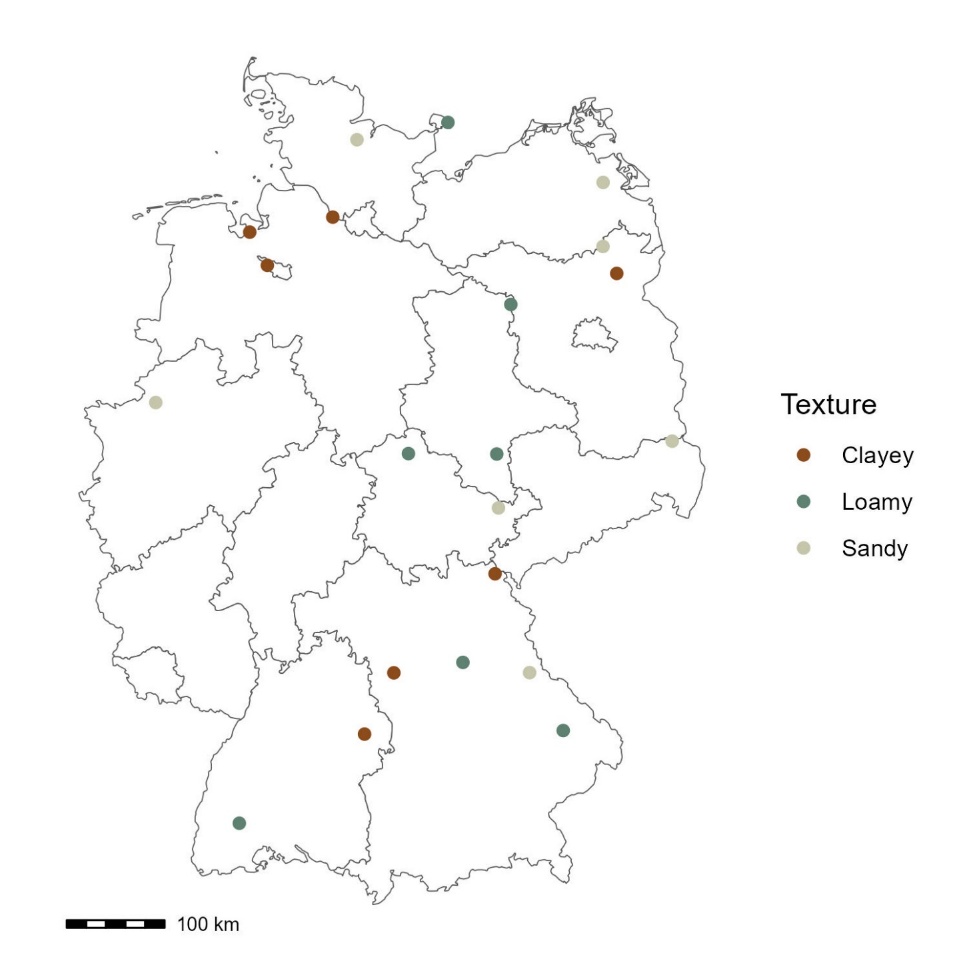


**Supplementary Figure S8.** *Distribution of selected soil samples across Germany, categorized by texture type: clayey, loamy, and sandy****.***


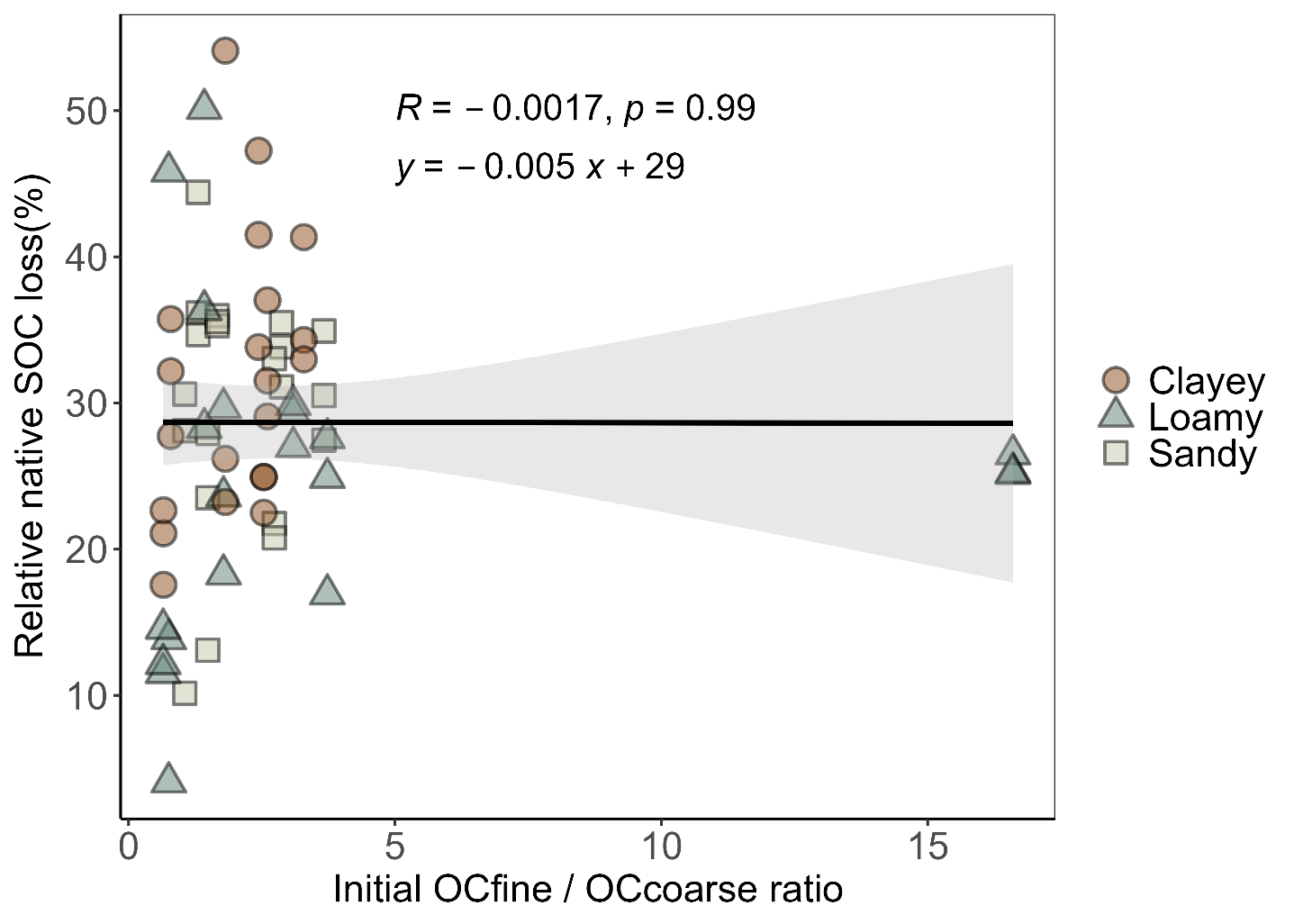


**Supplementary Figure S9.** Relationship between initial OC_fine_/OC_coarse_ ratio and relative native SOC loss after two years of incubation.
